# High‐Pressure Synthesis of Antimony Nitride Sb_3_N_5_ and Polynitride Sb_2_N_8_ Featuring Single‐Bonded N_8_^10−^ Chains

**DOI:** 10.1002/anie.202522652

**Published:** 2026-01-01

**Authors:** Lukas Brüning, Nityasagar Jena, Elena Bykova, Konstantin Glazyrin, Ievgeniia Iermak, Stella Chariton, Vitali B. Prakapenka, Igor A. Abrikosov, Maxim Bykov

**Affiliations:** ^1^ Institute for Inorganic and Analytical Chemistry Goethe University Frankfurt Frankfurt am Main 60438 Germany; ^2^ Department of Physics Chemistry and Biology (IFM) Linköping University Linköping SE‐58183 Sweden; ^3^ Institute of Geosciences Goethe University Frankfurt Frankfurt am Main 60438 Germany; ^4^ FS‐PETRA‐D Deutsches Elektronen‐Synchrotron (DESY) Hamburg 22607 Germany; ^5^ Applications Department Oxford Instruments Ulm 89081 Germany; ^6^ Center for Advanced Radiation Sources The University of Chicago Lemont Illinois 60439 USA

**Keywords:** Diamond Anvil Cell, High‐Pressure High‐Temperature, Nitride, Polynitride

## Abstract

High‐pressure high‐temperature synthesis in laser‐heated diamond anvil cells provides a direct pathway to stabilize polymeric and oligomeric nitrogen units, often inaccessible under ambient conditions due to the stability of the N_2_ molecule. Here we report the high‐pressure reactivity of antimony with molecular nitrogen, leading to the discovery of two distinct binary nitrides. At megabar pressures, we synthesized and structurally characterized *mP*20‐Sb_2_(N_8_), the first pnictogen oligonitride containing unprecedented twisted single‐bonded (N_8_)^10−^ chains. The compound was identified by single‐crystal x‐ray microdiffraction and Raman spectroscopy, supported by density functional theory calculations. At lower pressures of ∼ 50 GPa, we observed the formation of *oC*32‐Sb_3_N_5_, complementing recent reports of this phase by the studies of its compressional behavior and stability field. These results significantly expand the interpnictogen chemistry and demonstrate the ability of antimony to stabilize unusual extended nitrogen fragments at high pressures.

## Graphical abstract

High‐pressure reactions between Sb and molecular nitrogen in a laser‐heated diamond anvil cell led to the formation of *oC*32‐Sb_3_N_5_ (51(1) GPa) and *mP*20‐Sb_2_(N_8_), (> 100 GPa). Both phases were identified by single‐crystal x‐ray microdiffraction and are fully corroborated by density functional theory calculations. *mP*20‐Sb_2_(N_8_) is the first oligonitride to contain previously unknown twisted single‐bonded (N_8_)^10−^ chains, whose characteristic vibrations were additionally identified by Raman spectroscopy.

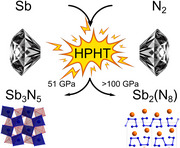

Interest in nitrogen‐based materials has grown in the recent decades due to their diverse properties and applications that include ultrahigh hardness and incompressibility,^[^ ^1^, ^2^, ^3^, ^4^, ^5^ ^]^ use in LED devices,^[^ ^6^, ^7^ ^]^ use as electrode materials in storage devices,^[^ ^8^ ^]^ use in water splitting,^[^ ^9^ ^]^ and use as refractory materials.^[^ ^10^ ^]^ However, the stability of the dinitrogen molecule N≡N at ambient conditions is a fundamental challenge for nitride chemistry.^[^ ^11^ ^]^ Conversely, many metastable dinitrides, oligonitrides, and polynitrides possess energy rich N─N bonds and may undergo exothermic fast release of N_2_ gas under thermal activation. Therefore, the syntheses of azide salts featuring (N_3_^−^), *cyclo*‐pentazolates (N_5_^−^) salts, or nitrogen allotropes are gaining in relevance for the search of environmentally‐friendly energy storage materials.^[^ ^12^, ^13^, ^14^, ^15^ ^]^ While most of the classical syntheses of azides and *cyclo‐*pentazolates are performed in a kinetically‐controlled regime at low temperatures to prevent their decomposition, a straightforward high‐pressure synthesis pathway exists, because pressure alone can shift the chemical equilibrium toward oligomerized and polymerized nitrogen compounds. Molecular nitrogen exhibits transformation to single‐bonded allotropes at megabar pressure (>110 GPa).^[^ ^16^, ^17^, ^18^, ^19^, ^20^ ^]^ Introduction of electron‐donating elements into the growing polymeric nitrogen network allows to stabilize various poly‐ and oligo‐nitride units, which not only provides a fundamental insight into the chemistry of nitrogen at extreme conditions, but also potentially allows to preserve these compounds upon pressure quenching. The state‐of‐the‐art method for synthesizing materials under these conditions is the laser‐heated diamond anvil cell (LH‐DAC) technique, which allows reaching pressures up to 1 TPa and temperatures up to 10000 K,^[^ ^21^, ^22^ ^]^ and enables in‐situ single‐crystal x‐ray diffraction (sc‐XRD) and spectroscopic investigations of synthesis products.^[^ ^23^ ^]^

Upon pressure increase, there is a tendency for the formation of binary nitrides and polynitrides with progressively increasing amount of nitrogen.^[^ ^24^ ^]^ Many high‐pressure compounds are reported in which a metal is reducing the N_2_ molecule to (N_2_^x−^) anions with broadly variable negative charges.^[^ ^2^, ^25^, ^26^, ^27^, ^28^, ^29^, ^30^, ^31^, ^32^, ^33^ ^]^ However, at pressures above 100 GPa, nitrogen tends to form linear 1D chains. Fundamental examples are the polyacetylene‐like anions [N_4_^2−^]*_n_* with delocalized^[^ ^34^, ^35^, ^36^, ^37^, ^38^ ^]^ and localized^[^ ^39^ ^]^ π‐electron systems, as well as single‐bonded chains [N_4_^4−^]*_n_*.^[^ ^40^ ^]^

Other less common motifs are anionic branched nitrogen chains,^[^ ^41^, ^42^ ^]^ nitrogen double helices,^[^ ^43^ ^]^ or 2D layered networks.^[^ ^44^ ^]^ Intermediate species, classified as oligonitrides, can also be stabilized by proper choice of cation as reported for *cis*‐tetranitrogen (N_4_^4−^) at pressures between 60 and 100 GPa.^[^ ^35^, ^40^ ^]^ The next higher analogues for linear oligonitride chains are (N_6_^6−^) and (N_8_^6−^) with single‐ and double bonds, which were recently reported in scandium polynitrides.^[^ ^44^ ^]^ In addition, there is a variety of cyclized nitrogen rings achieved by high‐pressure high‐temperature (HPHT) synthesis like pentazolate anions (N_5_^−^),^[^ ^28^, ^45^, ^46^, ^47^, ^48^ ^]^ hexazine‐based anions,^[^ ^49^, ^50^, ^51^ ^],^ or anionic (N_18_)‐macrocycles.^[^ ^43^ ^]^

Until recent years, crystalline binary nitrides of pnictogens (Group 15) were rather unexplored except for *α*‐P_3_N_5_, *β*‐P_3_N_5_, and *γ*‐P_3_N_5_.^[^ ^52^, ^53^, ^54^ ^]^ The pnictogen elements N, P, As, Sb, and Bi have the same valence shell electron configuration *s^2^p^3^*. Their common oxidation states are +5 and +3. The number of reported pnictogen nitrides increased rapidly due to HPHT experiments performed in LH‐DACs. For phosphorus, two polymorphs *α’*‐P_3_N_5_ and *δ*‐P_3_N_5_ were discovered alongside pyrite‐type PN_2_·(e^−^).^[^ ^55^ ^]^ In addition, pressure stabilized the formation of AsN and BiN, in which As and Bi exhibit a formal oxidation state of +3 as well as stereochemically active lone pairs.^[^ ^56^, ^57^ ^]^ Antimony nitrides were first discovered in the form of thin films produced by rapid thermal annealing and sputtering.^[^ ^58^, ^59^ ^]^ Bulk antimony nitride phases were proposed theoretically^[^ ^60^ ^]^ and the first crystalline classical antimony nitride, *oC*32‐Sb_3_N_5,_ was recently reported by Ceppatelli et al. in a HPHT synthesis.^[^ ^61^ ^]^ In this compound, antimony exhibits a formal oxidation state of +5, similar to phosphorus in P_3_N_5_ polymorphs. Recent studies demonstrated that the synthesis of novel pnictogen nitrides in DACs may be influenced by the presence of carbon from the diamond anvil, as we shown for the HPHT synthesis of nitridocarbonates of Sb and Bi.^[^ ^62^, ^63^ ^]^

This study focuses on the interpnictogen chemistry and, in particular, on the novel compounds in the Sb─N system over a wide pressure range. To the best of our knowledge, we characterized the first binary oligonitride stabilized by another pnictogen, namely *mP*20‐Sb_2_(N_8_) at megabar pressures. The (N_8_^10−^) fragment can be seen as tetramer of the dinitrogen molecule and as a single‐bonded oligiontride chain N_2x_ in general. It is experimentally characterized by sc‐XRD and Raman spectroscopy. Moreover, we observed the formation of the classical nitride *oC*32‐Sb_3_N_5_ at 51(1) GPa_._ We complement the recent study of Sb_3_N_5_ by an equation of state upon decompression, density functional theory (DFT) calculations and stability field of *oC*32‐Sb_3_N_5._

A LH‐DAC experiment was performed to investigate the reaction between antimony and nitrogen at pressures near the range where polymerized nitrogen is thermodynamically favored (for the detailed description, see Supporting Information sec. A‐B). A piece of antimony was compressed to approximately 100 GPa in a nitrogen atmosphere. Double‐sided laser heating of the antimony piece (Nd:YAG laser, *λ*  =  1064 nm, online set‐up GSECARS^[^ ^64^ ^]^) led to a pressure increase to 106(3) GPa. While most of the *bcc‐*Sb^[^ ^65^ ^]^ remained unreacted, a new phase formed at the contact area with nitrogen. Multigrain x‐ray diffraction data were collected at the sample areas, where the reaction had taken place. The package Domain Auto Finder (DAFi),^[^ ^66^ ^]^ implemented in the *CrysAlis^Pro^* Software, was able to identify several grains featuring similar primitive monoclinic Bravais lattices with the parameters *a *= 4.287(6) Å, *b *= 6.919(12) Å, *c *= 4.502(10) Å, and *β* = 92.40(15)° for the representative grain. Structure solution and refinement revealed the stoichiometry Sb_2_N_8_ and space group *P*2_1_/*n* for this crystal, where all atoms occupy general Wyckoff sites *4e* (No. 14–2, Tables S1–S3). This structure is reliably reproducible in geometry optimized calculations within DFT (see Tables S4 and S5, bond distances in brackets correspond to calculated values). Antimony is coordinated in a monocapped tetragonal antiprism by nitrogen atoms with Sb–N distances ranging from 2.005(14) Å (2.019 Å) to 2.252(11) Å (2.274 Å). The SbN_9_ polyhedron depicts the highest coordination number observed in any binary and ternary antimony nitrides.

Structure representations of *mP*20‐ Sb_2_N_8_ are visualized in Figure 1. Nitrogen atoms form 8‐membered chains, where 6 nonterminal nitrogen atoms form a planar backbone, while terminal nitrogen atoms are positioned out of plane. The N─N bond lengths range from 1.339(15) (1.352 Å) to 1.438(14) Å (1.412 Å), corresponding to the range of N─N single bonds.^[^ ^32^ ^]^ The structure can be visualized as alternating stacking of antimony atoms and layers of isolated N_8_ fragments along the crystallographic *b*‐axis. In the literature, this N_8_ fragment was theoretically predicted in the binary Sb─N system by Lian et al.,^[^ ^60^ ^]^ but their structure model is deviating from our experimentally observed one. We observe two distinguishable layers of N_8_ fragments, related by the *n* glide plane of the space group (see Figures 1 and S1). In contrast, the predicted structure is simpler, with only one distinguishable layer of N_8_ fragments and half formula units *Z* per unit cell.

**Figure 1:**
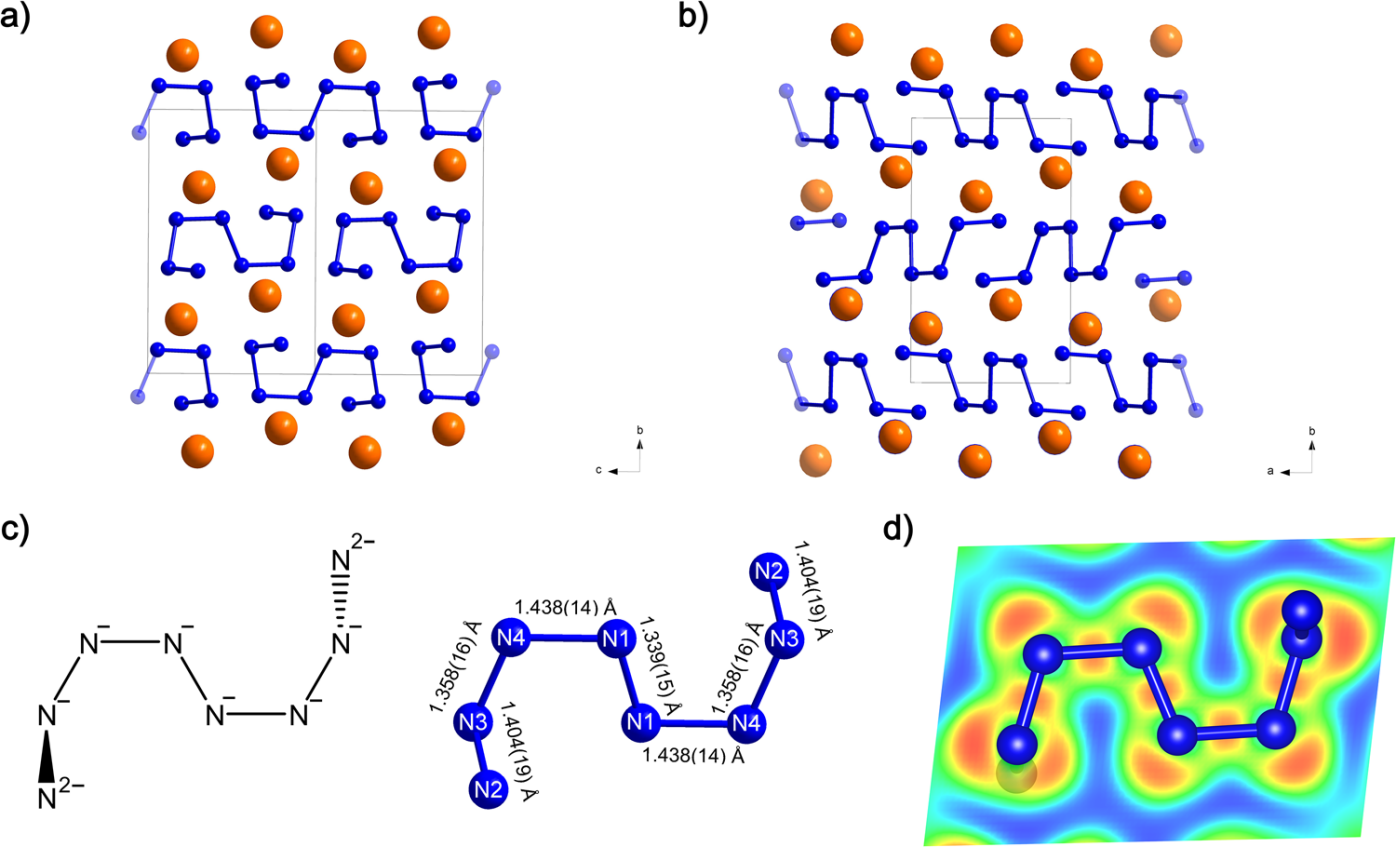
Crystal structure of *mP*20‐Sb_2_(N_8_) along a) the crystallographic *a*‐direction and b) along the crystallographic *c*‐direction with single‐bonded N_8_^10−^ chains (blue) between layers of antimony atoms (orange). c) Geometry, bond lengths, and skeleton formula of the (N_8_^10−^) oligonitrogen unit. d) 2D surface projection of the electron localization function (ELF) spanned by the six non‐terminal nitrogen atoms of the (N_8_^10−^) unit. The ELF is at an isosurface level of 0.8 e/Å^3^.

Bader charge analysis shows that Sb atoms act as electron donors for the N atoms in *mP*20‐Sb_2_(N_8_), with a substantial positive charge of +2.80 |e| at 120 GPa, while the N_8_ species adopt a charge of −1.11 |e| for terminal N_2_ atoms of the chain and −0.6,−0.61,−0.63 |e| for the non‐terminal N_3_, N_4_ and N_1_ atoms respectively (see Table S6).

The assumption of a single‐bonded fragment leads to N charges of −1 in the inner part of the chain and −2 for terminal N atoms, which is in very good agreement with the Bader charge analysis. Therefore, the resulting (N_8_^10−^) fragment is charge‐balanced by two Sb atoms in oxidation state +5. Electron localization function (ELF), as exemplified in Figure 1d, indicates the formation of intramolecular N─N directional covalent bonds, supporting this model. Antimony in oxidation state +5, as in the case of Sb_3_N_5_ (see Bader charges in Table S7) and SbCN_3_,^[^ ^62^ ^]^ is a reasonable assumption for elements from the pnictogen group. The oligonitride chain is different from the (N_8_^6−^) chain observed in Sc_2_(N_8_) at 78 GPa. In contrast to Sb_2_(N_8_), (N_8_^6−^) in Sc_2_(N_8_) possesses two double bonds with a bond length of 1.297 Å and represents a nearly planar chain.^[^ ^44^ ^]^ To the best of our knowledge, the single‐bonded (N_8_^10−^) chain has never been experimentally reported in terms of connectivity, charge, and geometry. There is one reported analogue for arsenic (As_8_^10−^) with a different chain geometry in Ca_2_As_3_.^[^ ^67^ ^]^

After the initial synthesis, the DAC sample was compressed to approximately 118 GPa and reheated, resulting in a pressure increase to 120(3) GPa and in the recrystallization and further formation of *mP*20‐Sb_2_(N_8_) from Sb and N_2_ as identified by sc‐XRD data (see Table S3) and a 2D‐PXRD map (see representative PXRD in Figure 2d).

**Figure 2:**
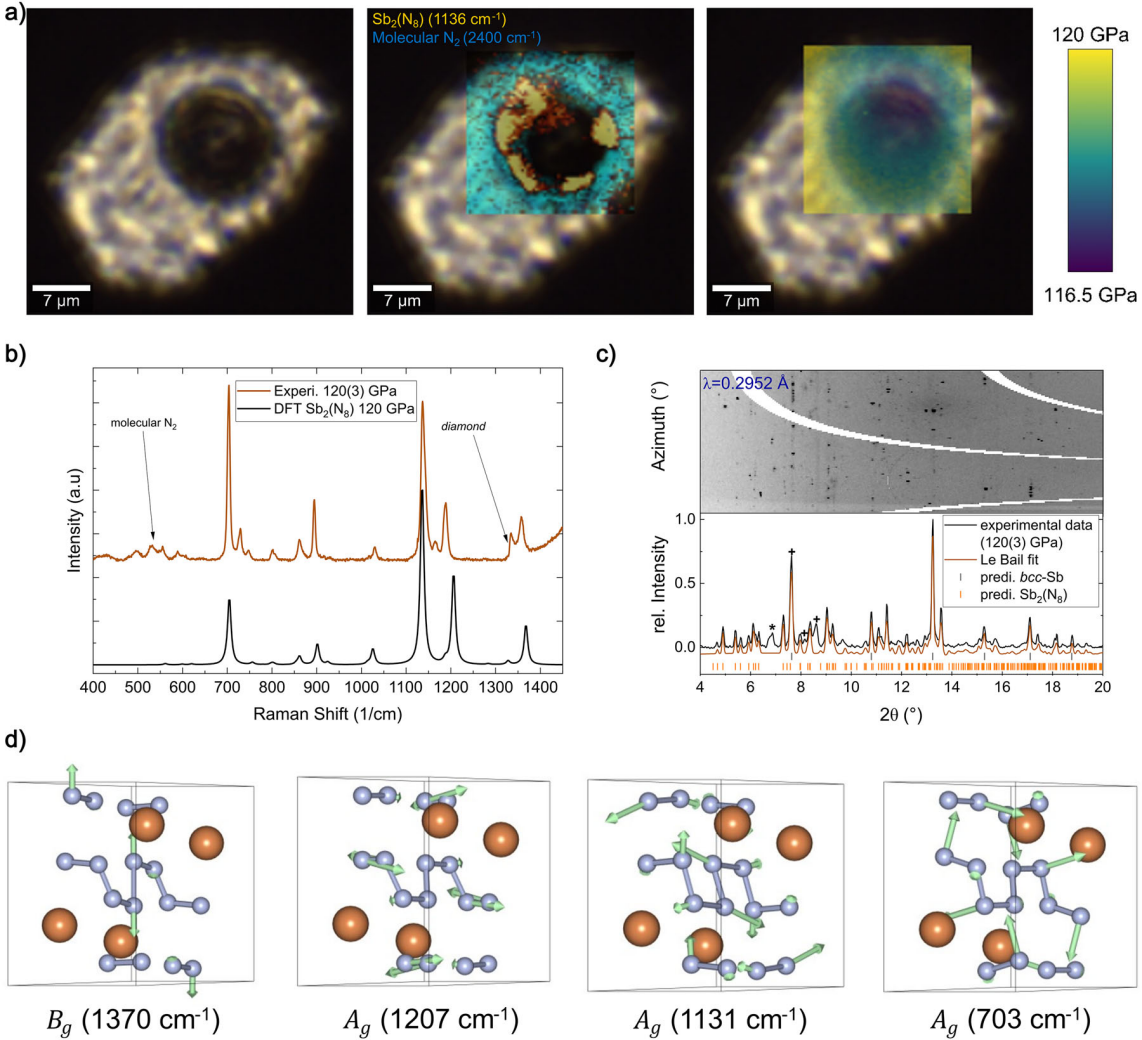
a) Microscope picture of the sample chamber at 120(3) GPa. The heated piece of antimony appears dark, and the phase distribution map indicates the formation of *mP*20‐Sb_2_(N_8_) at the edge of the piece. The phase distribution map was generated by plotting the intensity of the N_2_ stretching vibration band at ≈ 2400 cm^−1^ (cyan) and the *A_g_* vibration of *mP*20‐Sb_2_(N_8_) at 1136 cm^−1^ (yellow). Pressure gradient map was obtained by fitting the first derivative of the diamond Raman edge, as described in the Supporting Information, section D. b) Experimental Raman Spectrum of Sb_2_(N_8_) at 120 GPa in comparison with the DFPT calculated Raman spectrum. c) Azimuthally integrated x‐ray diffraction image, diffraction pattern, and its Le Bail fit from polycrystalline *mP*20‐Sb_2_(N_8_) and unreacted *bcc*‐Sb (*a, b, c* = 3.14 Å),^[^ ^65^ ^]^ at 120(3) GPa. * marks the (110) reflection from cg‐N.^[^ ^16^ ^]^ Reflections marked with + belong to the gasket material (*hcp*‐Re). d) Eigen vectors of the Raman active vibrations at selected frequencies corresponding to the Raman Spectrum of *mP*20‐Sb_2_(N_8_) (orange Sb, blue N) at 120 GPa.

We performed a 2D Raman scanning map (for details see Supporting Information sec. D) around the heated area. A rich Raman spectrum was observed at the edge of the antimony piece, where the reaction between Sb and N_2_ took place. The nearly complete absence of the N_2_ stretching vibration in the heated area indicates that the required thermal activation was reached either to polymerize nitrogen at megabar pressures^[^ ^20^ ^]^ or to involve it in the reaction with Sb. A phase distribution map was generated by plotting the background‐corrected intensity of the N_2_ vibration band at ≈ 2400 cm^−1^ and of a new signal at 1136 cm^−1^ (see Figure 2a). We collected a Raman spectrum in a range from 150 to 3000 cm^−1^ at the spot with the highest intensity of the new peak at 1136 cm^−1^, which appeared after the heating. Subsequently, Raman modes and their intensities of *mP*20‐Sb_2_(N_8_) were calculated using density functional perturbation theory (DFPT) at the Γ‐point and compared to the experimental spectrum (see Figure 2b). There is a very good agreement between the positions and intensities of the bands, which further validates the *mP*20‐Sb_2_(N_8_) structure model. Most known polynitrides exhibit anion‐driven metallicity and do not produce strong Raman signals. Exceptions are cyclized pentazolate anions^[^ ^28^, ^45^, ^47^ ^]^ and cyclized single‐bonded hexazine anions.^[^ ^51^ ^]^ This makes *mP*20‐Sb_2_(N_8_) the first chain oligonitride with a characterized Raman signature. Group theory predicts 15 Raman‐active modes with *A_g_* symmetry and 15 Raman modes with *B_g_* symmetry. For a deeper characterization of the (N_8_^10−^) chain, we created vibrational eigen vector images of the 4 most intense Raman active vibrations, as depicted in Figure 2c. At ambient conditions, the stretching vibration of the nitrogen dimer is proportional to the bond order, as described for binary nitrides in solid phases.^[^ ^29^, ^68^ ^]^ The characteristic Raman shift of the pernitride anion (N─N)^4−^ lies in the range of 800–1000 cm^−1^.^[^ ^25^, ^27^, ^69^ ^]^ However, group vibrations in polynitrides can have a broader range of energies. In case of *mP*20‐Sb_2_(N_8_), the intense group vibrations range from 703–1370 cm^−1^, which is similar to observed vibrations in other single‐bonded polynitrides like *cg*‐N,^[^ ^16^ ^]^ *bp*‐N,^[^ ^18^ ^]^ and WN_6_^[^ ^51^ ^]^ observed above megabar pressures. The *A_g_* vibration at 1131 cm^−1^ resembles an elongation of the entire N_8_ fragment, while the *B_g_* vibration at 1370 cm^−1^ is purely dominated by the stretching between N1─N1 at the center of the N_8_ chain. The other two *A_g_* vibrations at 703 and 1207 cm^−1^ correspond to out‐of‐plane movements and twisting.

First‐principles‐based DFT calculations were performed to investigate the crystal structure, lattice stability, and electronic properties of Sb_2_(N_8_). The generalized gradient approximation (GGA) with the Perdew–Burke–Ernzerhof (PBE) functional was employed for Sb_2_(N_8_) (see Supporting Information sec. E). The computed lattice parameters deviate by less than 2% from their experimental values (see Table S4). The pressure–volume relationship obtained from experiments shows excellent agreement with the calculated equation of state (EoS), well within the experimental pressure uncertainty of ±3 GPa. The DFT‐optimized lattice parameters at various scaled volumes were fitted using a third‐order Birch–Murnaghan EoS (see Figure 3a), which predicts a relatively low bulk modulus (K_0_) of ≈ 33 GPa and high K_0_’ of ≈ 8 for Sb_2_(N_8_). Figure 3b shows the phonon dispersion relations of *mP*20‐Sb_2_(N_8_) at 120 GPa. The absence of any imaginary phonon branches throughout the entire Brillouin zone (BZ) confirms the lattice dynamical stability of the compound at the synthesis pressure. The phonon modes below 10 THz are primarily dominated by Sb atom vibrations, while the high‐frequency optical modes arise mainly from N atom vibrations, as evident in the phonon density of states (PhDOS) and Raman modes (see Figure 2c).

**Figure 3:**
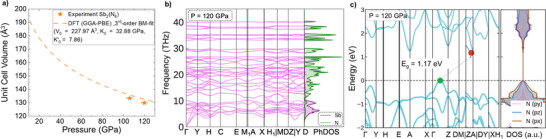
a) Calculated 3^rd^ order Birch–Murnaghan equation of state compared to the experimental unit cell volumes. b) Phonon dispersion relations at 120 GPa and phonon density of states (PhDOS) c) Electronic band structure of *mP*20‐Sb_2_(N_8_) at 120 GPa with an indirect semiconducting gap of 1.17 eV.

The electronic band structure of *mP*20‐Sb_2_(N_8_) at 120 GPa is shown in Figure 3c. The compound exhibits semiconducting behavior with an indirect band gap of ≈ 1.17 eV. The valence band maximum (VBM) lies along the midpoint of the Γ–Z k‐path, while the conduction band minimum (CBM) is located along the Z–A direction of the BZ. The orbital resolved electronic density of states (DOS) reveals that the nitrogen 2*p* orbitals dominate near the energy gap (see Figure S4). The orbital‐resolved DOS further indicates that the N(*p_x_*) orbitals contribute majorly at the valence band edge, followed by the N(*p_z_*) and N(*p_y_*) orbitals. The charge carrier effective masses calculated at the VBM/CBM band edges show a low electron effective mass over the hole mass (see Figures S4 and S5). Upon decompression, *mP*20‐Sb_2_(N_8_) retains its semiconducting character and exhibits no significant geometry changes of the N_8_^10−^ chain down to ≈ 40 GPa, below which it undergoes a transition to a metallic state (see Figures S3 and S4). Furthermore, the harmonic phonon dispersion relations indicate that Sb_2_(N_8_) remains dynamically stable down to ≈ 10 GPa, becoming dynamically unstable below this pressure (see Figures S5 and S6).

We have studied reactions between Sb and N_2_ in another experiment at lower pressure. Recent studies on the Sb─N and Sb─C─N system in LH‐DACs showed that a reaction between elemental Sb and nitrogen could not be observed at ≈ 15 and ≈ 20 GPa, but rather required pressures above 32 GPa.^[^ ^61^, ^62^ ^]^ In a similar approach, we compressed antimony to ≈ 50 GPa in a nitrogen atmosphere and induced a reaction with the online double‐sided laser heating (*T* = 2000(250)K) of the beamline P02.2 at Deutsches Elektronen‐Synchrotron (DESY) in Hamburg.^[^ ^23^ ^]^ Scanning the sample chamber with a 2 × 2 µm^2^ beam size indicated the formation of Sb_3_N_5_ (*Cmc*2_1_, No. 36–1, see Figure S2) as main product, which crystal structure was recently reported by Ceppatelli et al. at 32–35 GPa.^[^ ^61^ ^]^ We collected sc‐XRD data at suitable spots at synthesis pressure 51(1) GPa (lattice parameters *a *= 12.133(9) Å, *b *= 4.9919(8) Å, and *c *= 5.1328(9) Å). The structure contains two crystallographically distinct antimony atoms. Sb01 occupies a general Wyckoff site *8b* with an octahedral coordination by nitrogen atoms, while Sb02 occupies the Wyckoff site 4*a* in a trigonal prismatic coordination by nitrogen atoms. Consequently, the nitrogen atoms occupy tetrahedral voids in the antimony sublattice.

In order to study the stability field of *oC*32‐Sb_3_N_5_, we gradually decompressed the DAC to ambient pressure, performing sc‐XRD measurements at every pressure step (see Table S2), to obtain EoS data as depicted in Figure 4b,c. The pressure‐volume relationship obtained from our experiments shows an excellent agreement with the DFT calculations using the GGA‐PBEsol exchange‐correlation. The equilibrium unit cell volume of *oC*32‐Sb_3_N_5_ (*≈* 370 Å^3^), determined from the PBEsol calculations, closely matches the experimentally extrapolated volume using a 3rd‐order BM fit. The bulk modulus *K_0_* and its derivative *K_p_* for the experimental data are estimated to be 163(22) GPa and 6.5(13), respectively, which are in good agreement with the calculated values (see Figure 4b). This is significantly lower than that of δ‐P_3_N_5_ with *K*_0 _ =  299 GPa, where phosphorus is octahedrally coordinated.^[^ ^55^ ^]^ Considering that antimony is a softer cation than phosphorus due to its higher period, a lower bulk modulus is generally expected in binary compounds. The structure can be interpreted as interconnected SbN_6_‐octahedra and trigonal prisms (ratio 2:1). As visualized in Figure 4(a), the crystallographic *a*‐axis represents a stacking of two layers of corner sharing SbN_6_‐octahedra, followed by one layer of corner‐ and edge‐sharing SbN_6_‐prisms. Plotting the normalized unit cell parameter against pressure reveals that the *a*‐axis direction is more compressible than the other spatial directions. Analysis of the Sb–N distances (see Figure 4d) shows that the average bond distances within SbN_6_‐prisms and octahedra differ by *≈ *0.075 Å at synthesis pressure. However, they start to converge upon decompression and reach a difference of *≈ *0.012 Å at 4.6(10) GPa. Therefore, the anisotropic compression behavior of *oC*32‐Sb_3_N_5_ is due to the more compressible SbN_6_‐octahedra in comparison with the SbN_6_‐prisms that expand along the (*b,c*)‐plane.

**Figure 4:**
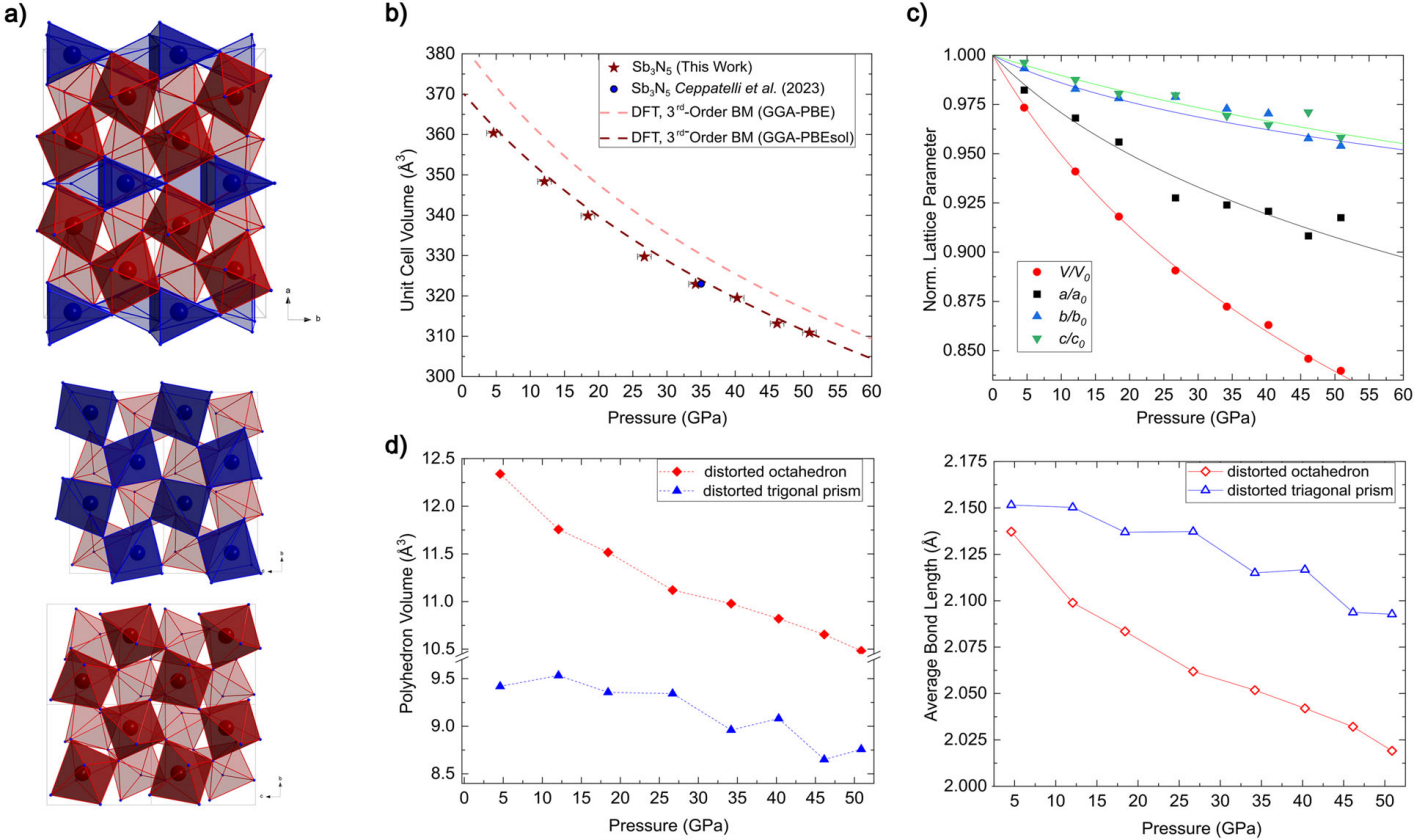
a) Crystal structure of *oC*32‐Sb_3_N_5_ represented by stacking SbN_6_‐octahedra (red) and trigonal prisms (blue). b) Pressure dependence of the unit cell volume of *oC*32‐Sb_3_N_5_ along with corresponding fits of the 3^rd^ order Birch–Murnaghan equation of state based on experimental data (*V*_0 _= 370(2), *K*_0_ = 163(22) GPa, *K′*_0_ = 6.5(13)) from this work and 1 pressure point from Ceppatelli et al.[61]), and DFT calculated volumes (GGA‐PBE, *V*_0 _= 382.56, *K*_0_ = 162.32 GPa, *K′*_0_ = 5.20; GGA‐PBEsol, *V*_0 _= 370.62, *K*_0_ = 184.17 GPa, *K′*_0_ = 5.11). c) Normalized lattice parameters plotted as function of pressure. Solid lines in c) correspond to the 3^rd^ order BM fit based on experimental values. d) Pressure dependences of polyhedron volumes and average Sb─N bond lengths of SbN_6_‐octahedra and trigonal prisms.

The phase proved to remain crystalline on decompression down to 4.6(10) GPa. Afterwards, we opened the DAC and exposed the sample for a short time to air. The crystallinity of the product decreased substantially and we were able to detect several tenths of crystallites in our ambient‐pressure dataset corresponding to crystalline Sb─I (*α*‐As type)^[^ ^65^ ^]^ and to an unknown phase (*mC*, *a ≈ *18.4 Å, *b ≈ *3.25 Å, *c ≈ *9.78 Å, and ß *≈* 101.4°). However, there is no clear evidence of remaining Sb_3_N_5_, but we cannot exclude a chemical reaction with oxygen or moisture after opening of the DAC. Harmonic phonon calculations using the PBEsol optimized geometries of *oC*32‐Sb_3_N_5_ do not show any imaginary phonon branches down to the ambient pressure (see Figure S7), suggesting that *oC*32‐Sb_3_N_5_ could potentially be preserved at ambient pressure (e.g., upon low‐temperature quenching in inert atmosphere). Figure S8 shows the electronic band structure of Sb_3_N_5_ at 50 GPa with an indirect semiconducting band gap of 1.33 eV. The VBM is located at the Y‐point of the BZ, whereas the CBM occurs at the *Γ*‐point. Upon decreasing the pressure to 5 GPa, *oC*32‐Sb_3_N_5_ undergoes a transition to a metallic state, while retaining the band edges at the Y‐ and Γ‐points, respectively (see Figures S8–S10).

Systematic probing of temperature‐induced reactions between antimony and excess nitrogen at different pressures resulted in the formation of *mP*20‐Sb_2_(N_8_) and *oC*32‐Sb_3_N_5_. *mP*20‐Sb_2_(N_8_) is the first pnictogen oligonitride with twisted single‐bonded (N_8_^10−^) chains, as identified by sc‐XRD and Raman spectroscopy, in combination with DFT calculations. Furthermore, both compounds contain antimony in the oxidation state +5, while a binary nitride with antimony in oxidation state +3 did not occur, unlike observed for its group neighbors As and Bi. Our experiments did not indicate any experimental evidence of a recoverable binary antimony nitride, even though *oC*32‐Sb_3_N_5_ is dynamically stable at ambient pressure according to our performed DFT calculations. However, we extended the experimental pressure range in which *oC*32‐Sb_3_N_5_ can be synthesized as main product to 32–50 GPa. To further investigate the thermodynamics of the antimony nitride formation, we calculated a convex hull diagram at pressures of 25 GPa, 50 GPa and 120 GPa (see Figure 5). Both *oC*32‐Sb_3_N_5_ and *mP*20‐Sb_2_(N_8_) lie below elementary Sb and N, indicating negative formation enthalpies at all three pressures steps. *mP*20‐Sb_2_(N_8_) was only synthesizable at above 100 GPa in our experiment. This indicates significant difference between the pressure ranges for the thermodynamic stability at 0 K and the pressure ranges required for the synthesis, which requires extremely high temperatures.

**Figure 5:**
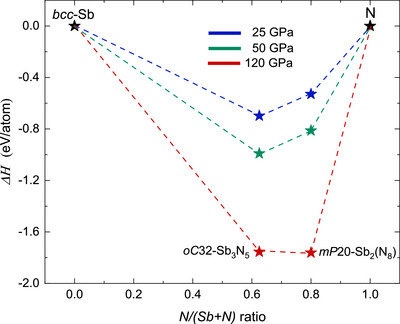
The convex hull diagram for the binary Sb─N system, based on experimentally observed structures at high‐pressure. The formation enthalpies of *mP*20‐Sb_2_N_8_ and *oC*32‐Sb_3_N_5_ were determined with respect to the *bcc*‐Sb and *∈*‐N_2_ at 25 and 50 GPa, whereas the enthalpies per atom at 120 GPa is evaluated with respect to *cg*‐N.

## Supporting Information

The authors have cited additional references within the Supporting Information.^[^ ^70^, ^71^, ^72^, ^73^, ^74^, ^75^, ^76^, ^77^, ^78^, ^79^, ^80^, ^81^, ^82^, ^83^, ^84^, ^85^, ^86^, ^87^, ^88^, ^89^, ^90^ ^]^ Deposition Numbers 2491510–2491511 (*mP*20‐Sb_2_(N_8_)) and 2491497–2491504 (*oC*32‐Sb_3_N_5_) contain the supplementary crystallographic data for this paper. These data are provided free of charge by the joint Cambridge Crystallographic Data Centre and Fachinformationszentrum Karlsruhe http://www.ccdc.cam.ac.uk/structures.

## Conflict of Interests

The authors declare no conflict of interest.

## Supporting information



Supporting Information

Supporting Information

## Data Availability

The data that support the findings of this study are available in the Supporting Information of this article.
