# Supplementary material for: High‐Pressure Synthesis of Antimony Nitride Sb3N5 and Polynitride Sb2N8 Featuring Single‐Bonded N8 10− Chains
Source: Angew Chem Int Ed Engl. 2026 Jan 1;65(6):e22652. doi: 10.1002/anie.202522652 (PMC12865237; doi:10.1002/anie.202522652)
Supplement: Supplementary file 1 — Supporting Information [file ANIE-65-e22652-s002.docx]

Supporting Information:

**High-Pressure Synthesis of Antimony Nitride Sb_3_N_5_ and Polynitride Sb_2_N_8_ featuring Single-Bonded N_8_^10-^ Chains**

Lukas Brüning*^[a]^, Nityasagar Jena*^[b]^, Elena Bykova^[c]^, Konstantin Glazyrin^[d]^, Ievgeniia Iermak^[e]^, Stella Chariton^[f]^, Vitali B. Prakapenka^[f]^ Igor A. Abrikosov^[b]^, and Maxim Bykov*^[a]^

[a] L. Brüning*, Prof. M. Bykov*
Institute for Inorganic and Analytical Chemistry, Goethe University Frankfurt
60438, Frankfurt am Main, Germany
E-mail: l.bruening@chemie.uni-frankfurt.de, maxim.bykov@chemie.uni-frankfurt.de

[b] Dr. N. Jena*, Prof. I. A. Abrikosov
Department of Physics, Chemistry and Biology (IFM), Linköping University
SE-58183, Linköping, Sweden

E-mail: nityasagar.jena@liu.se

[c] Prof. E. Bykova,
Institute of Geosciences, Goethe University Frankfurt
60438, Frankfurt am Main, Germany

[d] Dr. K. Glazyrin
Deutsches Elektronen-Synchrotron (DESY),
22607, Hamburg, Germany

[e] Dr. I. Iermak
Oxford Instruments,
89081, Ulm, Germany

[f] Dr. S. Chariton, Prof. V. B. Prakapenka
Center for Advanced Radiation Sources,
60439, Lemont, IL, USA

Contents

[Section A: DAC Preparation and Synthesis 2](#_Toc209615734)

[Section B: X-Ray Diffraction Methods 3](#_Toc209615735)

[Section C: SC-XRD Data 4](#_Toc209615736)

[Section D: Raman Spectroscopy 7](#_Toc209615737)

[Section E: DFT Calculations 8](#_Toc209615738)

[Section F: References 15](#_Toc209615739)

# Section A: DAC Preparation and Synthesis

Two independent experiments were performed for the synthesis of binary antimony nitrides, using BX90 diamond anvil cells (DACs) with Boehler-Almax type diamonds (100 µm culet for the 1^st^ experiment and 200 µm for the 2^nd^ experiment; opening angle for all diamond anvils was 60°). The preparation for both experiments was the same. A piece of Sb with a diameter of about 15 µm (30 µm + Ruby sphere for the 2^nd^ Experiment) was placed into a 50 µm (100 µm for 2^nd^ Experiment) hole in a Re gasket, which was preindented to a thickness of 24(3) µm. The diamond anvil cells were closed under liquid nitrogen atmosphere. Nitrogen served both as a pressure transfer medium (PTM) and as a reaction agent.

The 1^st^ DAC was precompressed to approximately 100 GPa and the antimony piece was double-sided laser heated above >3300 K with a Nd:YAG laser of wavelength 1064 nm. The pressure increased to 106(3) GPa, while most of the antimony occurred as unreacted, elementary, *bcc*-Sb^[1]^. At the edge of the heated spot, the minor phase of polycrystalline Sb_2_(N_8_) formed as a synthesis product. The DAC was then compressed to approximately 115 GPa and laser heated again (>3300 K, 120(3) GPa), which led to the recrystallization of and further conversion to Sb_2_(N_8_). Further attempts to increase the pressure resulted in premature failure of the diamond anvils and the end of this LH-DAC experiment.

The DAC of the 2^nd^ experiment was compressed to approximately 50 GPa and the antimony piece was double-sided laser heated to 2000(250) K with a Nd:YAG laser of wavelength 1064 nm. After heating, the pressure increased to 50.9(10) GPa and XRD diffraction images at the heated spot indicate the emergence of sharp reflections corresponding to multi-crystalline Sb_3_N_5_. The procedure for data acquisition is described in the section of XRD. The DAC was gradually decompressed to ambient pressure and Sb_3_N_5_ phase was remeasured with sc-XRD to obtain EoS-data.

The pressure for the 2^nd^ experiment was determined from the fluorescence spectra of chromium-doped ruby^[2,3]^ and the error is estimated to be around 1 GPa. The pressure in the 1^st^ DAC experiment was determined from the EoS of *hcp*-Re (Gasket)^[4]^ and the Raman edge. The uncertainty is estimated to be higher with 3 GPa (see Figure 2(a)).

# Section B: X-Ray Diffraction Methods

The synthesis products in our LH-DAC experiments are of crystalline nature, with submicron dimensions. The 1^st^ DAC was studied with synchrotron X-Ray diffraction at the beamline 13 ID-D (APS Chicago, λ≈0.2952 Å, Pilatus 1M CdTe flat panel detector)^[5]^ and the 2^nd^ DAC was studied at the beamline P02.2 (DESY Hamburg, λ≈0.2903 Å, Perkin Elmer XRD1621 flat panel detector).^[6,7]^ The beam diameters of the X-ryas were about 2 µm for both beamlines. The used setup for double-sided laser heating and its temperature determination from thermal emission is similar at both beamlines and described in literature.^[7]^

At synthesis pressure and selected pressure points during decompression, the sample areas were scanned with the X-ray beam to create a 2D‑PXRD grid. At selected points of the grid, we collected single-crystal data with a narrow 0.5° scanning step from -30° to +30° rotation angle ω.

Diffraction data analysis up to reflection data file was performed with the CrysAlisPro software and the integrated Domain Auto Finder (DAFi) program.^[8]^ DAFi sorts reflections from the peak table into groups, which presumably originate from the same grain of the typical polycrystalline dataset.

The resulting crystal lattice and orientation was used for final data integration with typical settings for high-pressure data sets. The resulting *hkl* file was used to refine a structure model within the OLEX2 interface.^[9]^ ShelXT^[10]^ was used for generating a suitable structure model and ShelXL^[10]^ was used for least squares refinement of the structure model. For Sb_2_(N_8_) at 120(3) GPa, a representative raw image of the 2D-PXRD map was azimuthally integrated to obtain a textured powder pattern using Dioptas.^[11]^ The Le Bail fit of the PXRD was simulated using JANA2006 with Gauß-type functions.^[12]^ The structures were visualized with Diamond. Polynator^[13]^ was used to calculate the deviation between occurring polyhedra in synthesized crystal structures and well defined geometrical bodies. Least-squares fitting of EoS parameters were performed with the program EosFit7.^[14]^

# Section C: SC-XRD Data

**Table S1.** Crystallographic data of Sb_2_(N_8_) (*P*2_1_*/n*) at both pressure steps. Single-crystal measurements were carried out with a synchrotron source of λ≈0.2952 Å wavelength and a Pilatus 1M CdTe flat panel detector (beamline 13 ID-D, APS).

| **Structure** | | **Sb_2_(N_8_)** | **Sb_2_(N_8_)** |
| --- | --- | --- | --- |
| **Pressure (GPa)** | | **106(3)** | **120(3)** |
| *a, b, c* (Å) | | 4.287(6), 6.919(12),  4.502(10) | 4.258(3), 6.877(4),  4.438(6) |
| *β* (°) | | 92.40(15) | 91.67(9) |
| *V* (Å^3^) | | 133.4(4) | 129.9(2) |
| µ (mm^−1^) | | 10.68 | 10.97 |
| *R_int_* | | 0.028 | 0.023 |
| (sin θ/λ)_max_ (Å^−1^) | | 0.855 | 0.865 |
| *R[F^2^ > 2σ(F^2^)], wR(F^2^), S* | | 0.037, 0.097, 1.19 | 0.042, 0.113, 1.08 |
| No. of measured, independent and observed [*I > 2σ(I)]* reflections | 344, 230, 188 | | 343, 233, 195 |
| No. of parameters | | 26 | 26 |
| *Δρ_max_*, *Δρ_min_* (e Å^−3^) | | 1.64, −1.55 | 2.57, −2.38 |

**Table S2.** Crystallographic data of Sb_3_N_5_ (*Cmc*2_1_) at different pressures. Single-crystal measurements were carried out with a synchrotron source of λ≈0.2903 Å wavelength and a Perkin Elmer XRD1621 flat panel detector (beamline P02.2, DESY).

| **Structure** | **Sb_3_N_5_** | **Sb_3_N_5_** | **Sb_3_N_5_** | **Sb_3_N_5_** |
| --- | --- | --- | --- | --- |
| **Pressure (GPa)** | **50.9(10)** | **46.1(10)** | **40.3(10)** | **34.2(10)** |
| *a, b, c* (Å) | 12.133(9), 4.9919(8), 5.1328(9) | 12.011(5), 5.0119(10), 5.2018(11) | 12.176(9), 5.078(2), 5.1671(19) | 12.218(7), 5.0906(10), 5.1922(9) |
| *V* (Å^3^) | 310.9(2) | 313.14(15) | 319.5(3) | 322.9(2) |
| µ (mm^−1^) | 13.13 | 13.04 | 12.78 | 12.64 |
| *R_int_* | 0.038 | 0.023 | 0.087 | 0.034 |
| (sin θ/λ)_max_ (Å^−1^) | 1.006 | 1.026 | 1.011 | 1.017 |
| *R[F^2^ > 2σ(F^2^)], wR(F^2^), S* | 0.060, 0.148, 1.25 | 0.043, 0.080, 1.07 | 0.057, 0.144, 1.06 | 0.062, 0.162, 1.04 |
| No. of measured, independent and observed [*I > 2σ(I)]* reflections | 656, 473, 404 | 666, 511, 459 | 651, 524, 468 | 665, 497, 453 |
| No. of parameters | 27 | 35 | 27 | 28 |
| *Δρ_max_*, *Δρ_min_* (e Å^−3^) | 3.53, −4.28 | 2.45, −2.38 | 4.76, −4.09 | 4.42, −4.80 |
|  |  |  |  |  |

| **Structure** | **Sb_3_N_5_** | **Sb_3_N_5_** | **Sb_3_N_5_** | **Sb_3_N_5_** |
| --- | --- | --- | --- | --- |
| **Pressure (GPa)** | **26.7(10)** | **18.4(10)** | **12.1(10)** | **4.6(10)** |
| *a, b, c* (Å) | 12.266(7), 5.122(3), 5.2485(17) | 12.6416(19), 5.1186(5), 5.2525(5) | 12.8026(8), 5.1434(4), 5.2904(8) | 12.99(3), 5.1980(17), 5.337(2) |
| *V* (Å^3^) | 329.7(3) | 339.87(7) | 348.37(6) | 360.3(10) |
| µ (mm^−1^) | 12.38 | 12.01 | 11.72 | 11.33 |
| *R_int_* | 0.063 | 0.021 | 0.012 | 0.083 |
| (sin θ/λ)_max_ (Å^−1^) | 1.022 | 1.01 | 1.038 | 1.027 |
| *R[F^2^ > 2σ(F^2^)], wR(F^2^), S* | 0.075, 0.154, 1.01 | 0.040, 0.129, 1.35 | 0.021, 0.053, 1.04 | 0.067, 0.151, 0.95 |
| No. of measured, independent and observed [*I > 2σ(I)]* reflections | 699, 521, 391 | 707, 550, 512 | 741, 552, 533 | 742, 515, 368 |
| No. of parameters | 27 | 27 | 27 | 27 |
| *Δρ_max_*, *Δρ_min_* (e Å^−3^) | 4.46, −5.52 | 3.96, −3.63 | 1.09, −1.59 | 3.22, −5.28 |
|  |  |  |  |  |


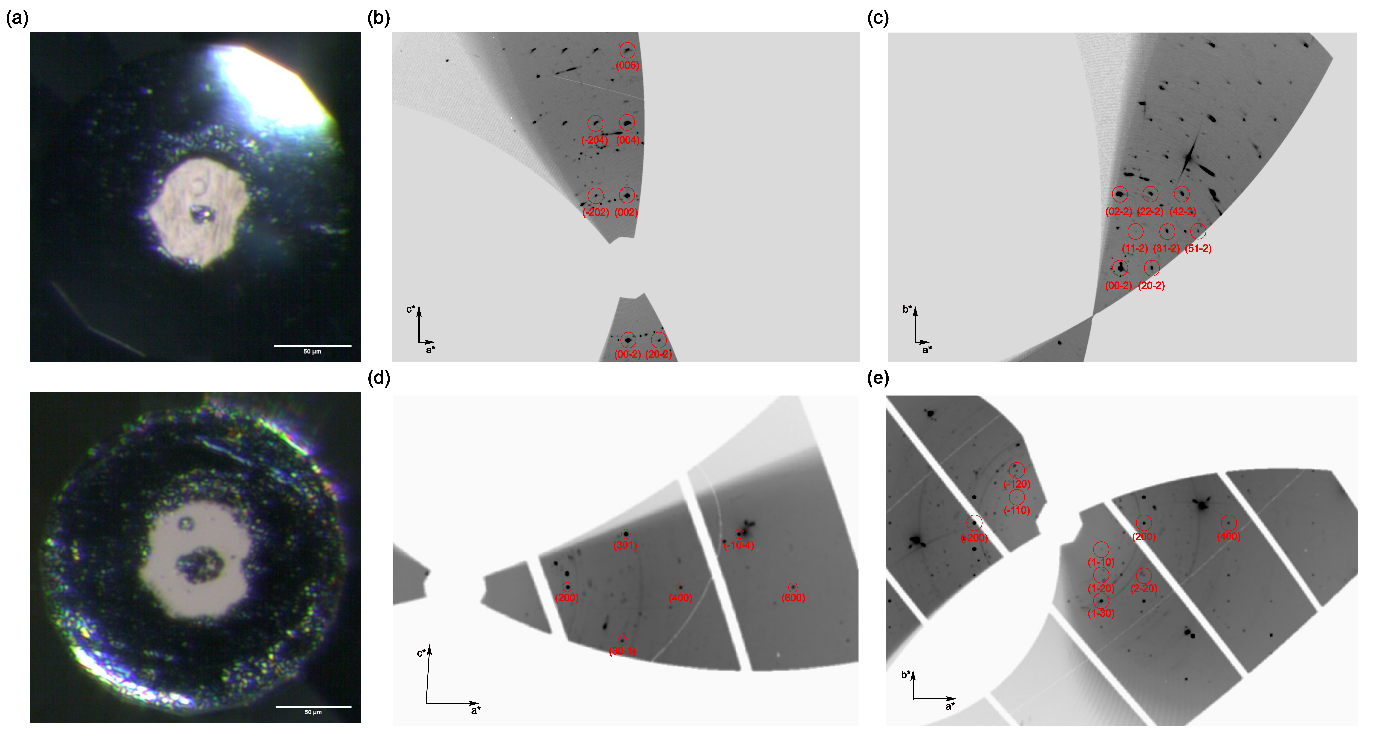


**Figure S1: (a)** Bright-field microscope image of the sample chamber (2^nd^ Experiment) at approximately 50 GPa before laser-heating and bright-field microscope image of the sample chamber after laser-heating, and decompression to 4.6(10) GPa. **(b)** (h0l)-reciprocal lattice plane of Sb_3_N_5_ (2^nd^ Experiment) with indexed reflections fulfilling l=2n (c-glide plane perpendicular to [010] + screw axis along [001]). **(c)** (hk-2)-reciprocal lattice plane of Sb_3_N_5_ (2^nd^ Experiment) with indexed reflections fulfilling h + k = 2n (C-centered lattice). **(d)** (h0l)-reciprocal and **(e)** (hk0)-reciprocal lattice planes of Sb_2_(N_8_) (1^st^ Experiment) at 106(3) GPa with indexed reflections fulfilling h+l=2n and h=2n (2 out 4 depicted reflection conditions for space group P2_1_/n (No. 14-2)).

**Table S3**: Fractional coordinates and Wyckoff sites of Sb_2_(N_8_) (SG No.14-2) at 106(3) GPa.

| **Atom** | **Wyck.** | ***x/a*** | ***y/b*** | ***z/c*** | **U_eq._ (Å^2^)** |
| --- | --- | --- | --- | --- | --- |
| Sb1 | 4*e* | 0.4057(2) | 0.29518(11) | 0.30025(19) | 0.007(2) |
| N1 | 4*e* | -0.003(2) | 0.4109(15) | 0.442(2) | 0.0017(16) |
| N2 | 4*e* | 0.550(3) | 0.388(2) | 0.705(3) | 0.0079(19) |
| N3 | 4*e* | 0.287(3) | 0.3992(17) | -0.120(3) | 0.0042(17) |
| N4 | 4*e* | 0.184(3) | 0.5828(16) | -0.166(2) | 0.0050(18) |


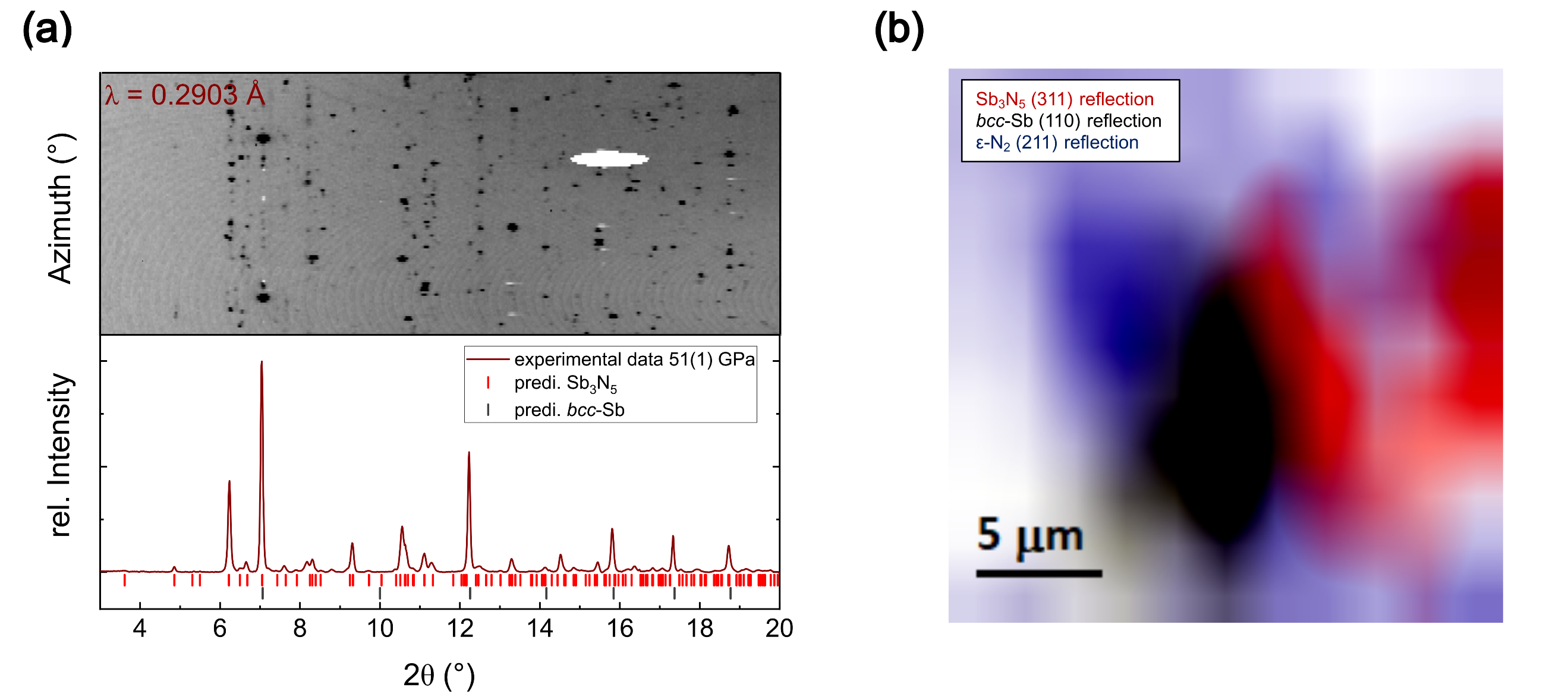


**Figure S2: (a)** Azimuthally integrated X-ray diffraction image and diffraction pattern from polycrystalline Sb_3_N_5_ (lattice parameter from sc-XRD data) and uncreated bcc-Sb (a, b, c = 3.335 Å)^[1]^ at 51(1) GPa after Synthesis. **(b)** 2D-PXRD map (as representative for a qualitatively phase distribution map). Red color code corresponds to the intensity of the (311) reflection of Sb_3_N_5_, black correspond to the intensity of the (100) reflection of bcc-Sb and blue correspond to the intensity of the (211) reflection of ε-N_2_.

# Section D: Raman Spectroscopy

Raman spectroscopy studies were performed using the Raman imaging microscope *WITec alpha 300R Raman* from Oxford Instruments. The system resembles a confocal microscope equipped with an Olympus 50x/0.35 objective, a motorized stage for micrometer precise movements, and an Andor DR316B-LDC CCD detector. The excitation wavelength was 532 nm and the beam had a spot diameter of ≈ 0.8 µm with a confocal depth of ≈ 6 µm. The single spectrum at the best position was acquired with a laser power of 50 mW and averaging of 10 accumulations with an acquisition time of 10 seconds each. We used a grating with 1200 g/mm (BLZ 500 nm).

The 2D Raman scanning map had the dimension 20 x 20 µm^2^ with 0.333 µm step size and 1 second acquisition time. A different grating was used (300 g/mm, BLZ 500 nm) for signal separation. For the phase distribution map, we plotted the intensity of the maxima around ≈ 1136 cm^-1^ (*A_g_*, Sb_2_(N_8_)) and ≈ 2400 cm^-1^ (N_2_ stretching vibration) and color coded the intensity against their position in the sample chamber. To produce a pressure gradient map, we first determined the positions of the high-frequency edge of the diamond Raman band at each point of the map by taking the first derivative of the spectrum and determining their maxima. These positions of these maxima were converted to pressure, using the pressure-frequency relationship of the stressed diamond Raman band provided by *Y. Akahama* and *H. Kawamura*.^[15]^ Cosmic ray removal, background subtraction and visualization of the Raman data were performed within the provided software by Oxford Instruments.

# Section E: DFT Calculations

The first-principles based electronic structure calculations were performed within the framework of density functional theory (DFT), as implemented in the Vienna Ab Initio Simulation Package (VASP, version 6.4.3).^[16,17]^ In plane wave DFT, the electronic wave functions were expanded in plane waves using the projector-augmented-wave (PAW) method.^[18,19]^ The generalized gradient approximation (GGA) with the Perdew–Burke–Ernzerhof (PBE) functional^[17]^ and the revised PBE for solids (PBEsol) functional^[20]^ were employed for calculating the exchange–correlation energies. The PAW potentials were taken from the VASP PBE potential library, with five valence electrons of configuration 5*s^2^*5*p^3^* for Sb and five valence electrons of configuration 2*s^2^*2*p^3^* for N.

The Monkhorst–Pack scheme^[21]^ was used for the k-point integration with grid densities of 16×7×11 for Sb_2_(N_8_) and 10×10×9 for Sb_3_N_5_. A plane-wave energy cutoff of 600 eV was chosen, with convergence criteria of 1 meV per atom for energy and 1 meV/Å per atom for forces. For self-consistent energy minimization, a convergence threshold of 10^-6^ eV was set for the free energy minimization. Variable-cell relaxations, including both lattice parameters and atomic positions, were performed on the experimental structures using the conjugate-gradient (CG) optimization scheme. Atomic coordinates and lattice vectors were optimized until the atomic forces were less than 10^-3^ eV/Å per atom and the residual Pulay stress was below 0.1 GPa.

Equation of state (EoS) calculations were performed using the variable-cell relaxations at different scaled volumes. A third-order Birch–Murnaghan (BM3) EoS was then fitted to the energy–volume data to obtain the pressure–volume relationship and EoS fitting parameters, including the equilibrium unit cell volume at ambient pressure, the bulk modulus, and its pressure derivative. The DFT-optimized lattice parameters and unit-cell volumes using the GGA-PBE functional showed good agreement with experiment for Sb_2_(N_8_), while GGA-PBEsol exhibited close agreement with the experimental pressure–volume relationship for Sb_3_N_5,_ as shown in the EoS plots in the main text.

Electronic structure calculations were performed using the GGA-PBE for Sb_2_(N_8_), and GGA-PBEsol for Sb_3_N_5,_ with the corresponding geometries. The tetrahedron method with Blöchl corrections was applied to obtain the ground-state electron density and the electronic density of states (DOS). The electron localization function (ELF) was computed on the same grid, and the electron density isosurfaces were visualized using the VESTA software.^[22]^

Phonon dispersion relations and Raman-active modes were calculated within the harmonic approximation using the PHONOPY code.^[23]^ The phonon band structures were obtained using the finite displacement method, with interatomic forces extracted from several symmetry-imposed 2×2×2 supercells for Sb_2_(N_8_) and Sb_3_N_5._The k-points for supercell calculations were adjusted according to the supercell lattice vectors, and the Brillouin zone (BZ) was sampled using the Monkhorst–Pack scheme. Raman modes were obtained using density functional perturbation theory (DFPT) at the Γ-point for the Sb_2_(N_8_) unit cell.

Finally, atomic charge transfer analysis was carried out using Bader’s charge density partitioning method.^[24]^ We incorporated both the core and valence charge densities for the Bader’s charge partitioning scheme. This approach ensures accurate evaluation of charge transfer between different atomic species and Wyckoff sites within a compound.

**Table S4**: Experimental lattice parameters for Sb_2_(N_8_) at 120 GPa and 106 GPa are compared with the DFT-optimized lattice parameters obtained using the GGA-PBE functional at the same pressures. The unit cell choice was changed to 14-1 for the experimental structure.

| **Crystal** | **Pressure (GPa)** |  | **a (Å)** | **b (Å)** | **c (Å)** | **α(°)** | **ß(°)** | **γ(°)** | **V (Å^3^)** | **ρ (g/cm^3^)** |  |
| --- | --- | --- | --- | --- | --- | --- | --- | --- | --- | --- | --- |
|  |  | **Space Group** |  |  |  |  |  |  |  |  |  |
|  |  | **(S.G.)** |  |  |  |  |  |  |  |  |  |
| *mP*20-SbN_4_ | 120 GPa | *P*2_1_/*c* (#14-1) | 4.438 | 6.877 | 6.06 | 90 | 135.4 | 90 | 129.865 | 9.093 |  |
|  |  |  |  |  |  |  |  |  |  |  |  |
|  |  |  |  |  |  |  |  |  |  |  |  |
|  | 106 GPa | *P*2_1_/*c* (#14-1) | 4.502 | 6.919 | 6.085 | 90 | 135.3 | 90 | 133.324 | 8.857 |  |
| **DFT** |  |  |  |  |  |  |  |  |  |  |  |
| GGA-PBE | 120 GPa | *P*2_1_/*c* (#14-1) | 4.282 | 6.907 | 6.089 | 90 | 132.66 | 90 | 132.427 | 8.917 |  |
| (Error %) |  |  | 3.50% | 0.40% | 0.50% | -- | 2% | -- | 2% | 1.93% |  |
| GGA-PBE | 106 GPa | *P*2_1_/*c* (#14-1) | 4.309 | 6.965 | 6.131 | 90 | 132.54 | 90 | 135.566 | 8.711 |  |
| (Error %) |  |  | 4.30% | 0.60% | 0.70% | -- | 2% | -- | 1.70% | 1.64% |  |

**Table S5**: Experimental lattice parameters for Sb_3_N_5_ at 51 GPa, compared with DFT-optimized lattice parameters at 50 GPa and at 1 bar using the GGA-PBE and GGA-PBEsol exchange–correlation functionals. The EoS shows excellent agreement with the experimental pressure–volume relation when using the GGA-PBEsol functional, as discussed in the main text.

| **Crystal** | **Pressure** | **Space Group** | **a (Å)** | **b (Å)** | **c (Å)** | **α(°)** | **ß(°)** | **γ(°)** | **V (Å^3^)** | **ρ** |
| --- | --- | --- | --- | --- | --- | --- | --- | --- | --- | --- |
|  | **(GPa)** | **(S.G.)** |  |  |  |  |  |  |  | **(g/cm^3^)** |
| *oC*32-Sb_3_N_5_ **(exp.)** | 50.9(10) GPa | *Cmc*2_1_ (#36) | 12.133(9) | 4.9919(3) | 5.128(9) | 90 | 90 | 90 | 310.9(2) | 9.301 |
| **DFT** |  |  |  |  |  |  |  |  |  |  |
| GGA-PBE | 50 GPa | *Cmc*2_1_ (#36) | 12.075 | 5.054 | 5.190 | 90 | 90 | 90 | 316.73 | 9.128 |
| GGA-PBEsol | 50 GPa | *Cmc*2_1_ (#36) | 11.980 | 5.031 | 5.169 | 90 | 90 | 90 | 311.61 | 9.279 |
| GGA-PBE | 1 bar | *Cmc*2_1_ (#36) | 13.358 | 5.281 | 5.420 | 90 | 90 | 90 | 382.34 | 7.562 |
| GGA-PBEsol | 1 bar | *Cmc*2_1_ (#36) | 13.126 | 5.241 | 5.382 | 90 | 90 | 90 | 370.35 | 7.807 |

**Table S6**: Bader charges on cations and anions in Sb_2_(N_8_) at 106 GPa and 120 GPa, respectively.

| **Bader Charges** | **Sb_2_(N_8_)** | **Wyckoff positions** | **Charges** | **Sb_2_(N_8_)** | **Wyckoff positions** | **Charges** |
| --- | --- | --- | --- | --- | --- | --- |
|  | **(P = 106 GPa)** |  |  | **(P = 120 GPa)** |  |  |
| Cations | Sb | 4*e* | 2.91 | Sb | 4*e* | 3.02 |
| Anions | N1 | 4*e* | -0.63 | N1 | 4*e* | -0.63 |
|  | N2 | 4*e* | -1.07 | N2 | 4*e* | -1.11 |
|  | N3 | 4*e* | -0.6 | N3 | 4*e* | -0.63 |
|  | N4 | 4*e* | -0.61 | N4 | 4*e* | -0.63 |

| **Bader Charges** | **Sb_3_N_5_** | **Wyckoff positions** | **Charges** | **Sb_3_N_5_** | **Wyckoff positions** | **Charges** |
| --- | --- | --- | --- | --- | --- | --- |
|  | **(P = 5 GPa)** |  |  | **(P = 50 GPa)** |  |  |
| Cations | Sb1 | *8b* | 2.66 | Sb2 | 8*b* | 2.84 |
|  | Sb2 | *4a* | 2.36 | Sb2 | 4a | 2.52 |
| Anions | N1 | *4a* | -1.32 | N1 | 4*a* | -1.50 |
|  | N2 | *8b* | -1.52 | N2 | 8*b* | -1.60 |
|  | N3 | *8b* | -1.65 | N3 | 8*b* | -1.75 |

**Table S7**: Bader charges on cations and anions in Sb_3_N_5_ at 5 GPa and 50 GPa, respectively.

| **15 A_g_** | **15 B_g_** |
| --- | --- |
| **(cm^-1^)** | **(cm^-1^)** |
| 217.9967 | 317.6213 |
| 248.8887 | 325.1667 |
| 265.9502 | 369.0219 |
| 566.7119 | 601.7047 |
| 621.0916 | 693.7287 |
| 703.1007 | 729.6697 |
| 756.76 | 782.0177 |
| 803.318 | 798.2102 |
| 865.3363 | 858.1249 |
| 904.1604 | 926.343 |
| 1025.229 | 1015.286 |
| 1130.905 | 1136.808 |
| 1207.251 | 1187.343 |
| 1326.233 | 1281.325 |
| 1366.233 | 1369.878 |

**Table S8**: Calculated Raman active modes and the corresponding vibrational frequencies of Sb_2_(N_8_) at 120 GPa. Raman active modes: 15*A*_g_ + 15*B*_g_.


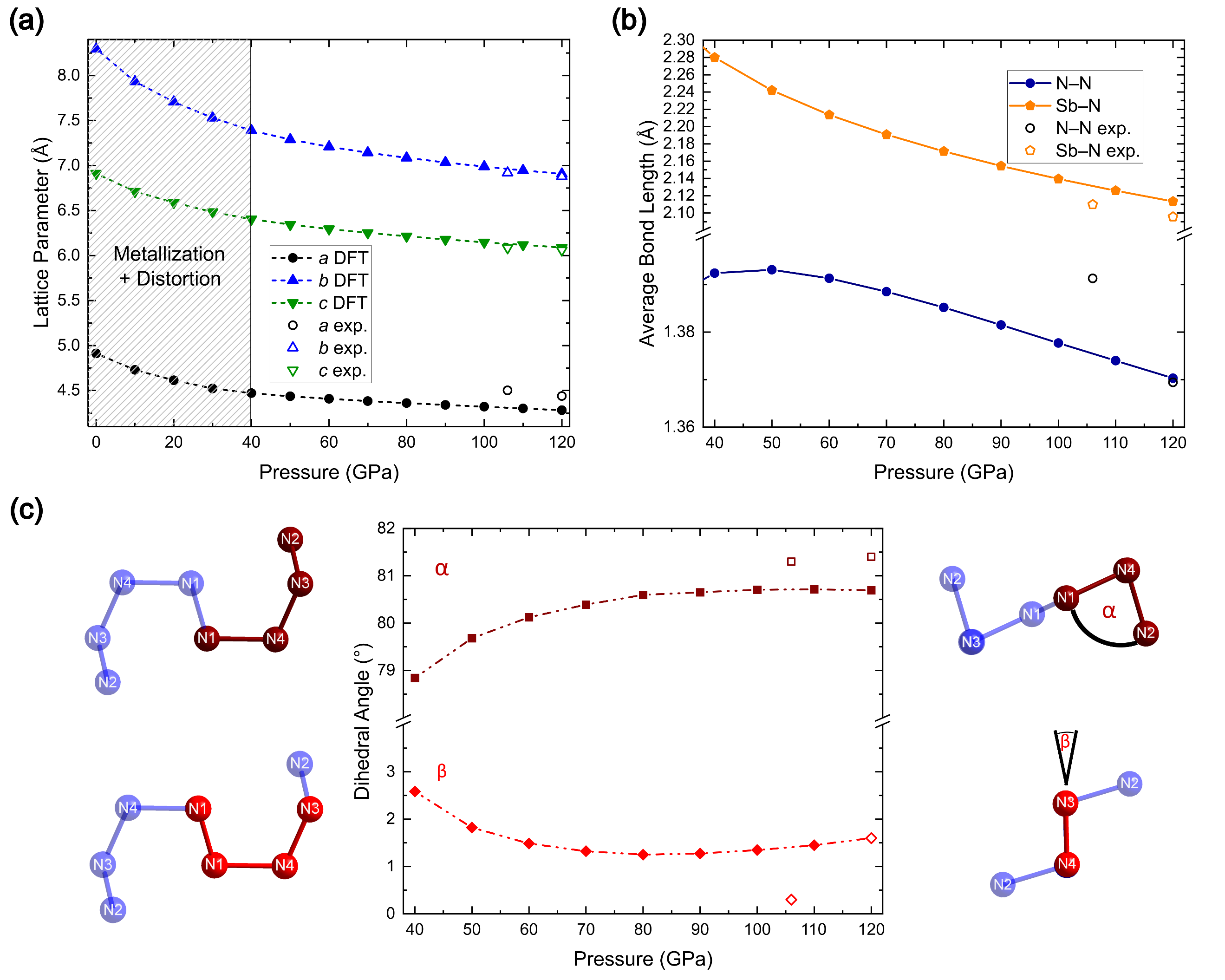


**Figure S3: (a)** Pressure-dependent lattice parameters of mP20-Sb_2_(N_8_) based on DFT geometry optimization (see Table S4 for DFT details). Below 40 GPa, the predicted structure model starts to significantly distort and undergoes metallization (see Figure S4). **(b)** Average N-N bond lengths within the N_8_^10-^ chain and average Sb-N bond lengths (considering monocapped tetragonal antiprismatic coordination) as functions of pressure. **(c)** Pressure dependence of dihedral angles in the N_8_^10-^ chain (N1-N4-N3-N2, and N1-N1-N4-N3), showing no significant distortions over a broad pressure range.


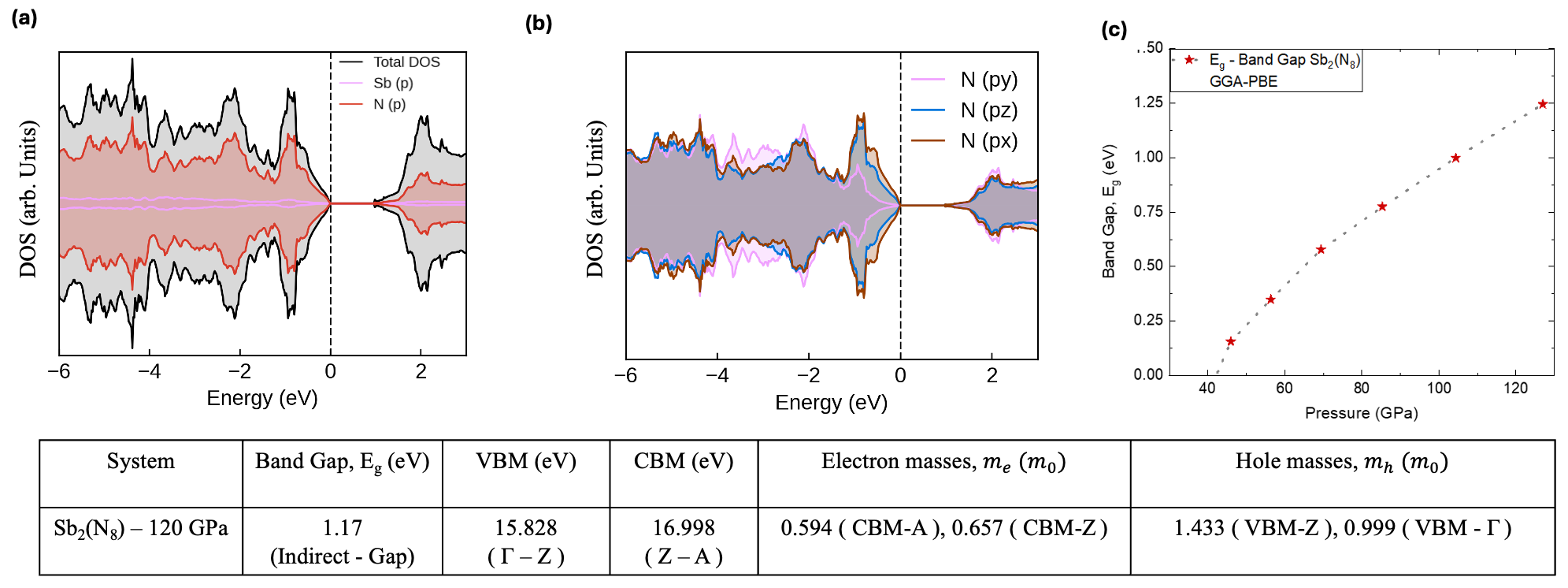


**Figure S4: (a)** Total and atom projected electronic density of states (DOS) at 120 GPa. **(b)** The orbital resolved DOS for nitrogen 2p-orbitals in Sb_2_(N_8_) at 120 GPa. **(c)** Pressure dependent indirect energy gap variations in Sb_2_(N_8_) using DFT calculations with GGA-PBE functionals. The table in the bottom panel shows the effective electron/hole masses at the band edges of Sb_2_(N_8_) at 120 GPa.


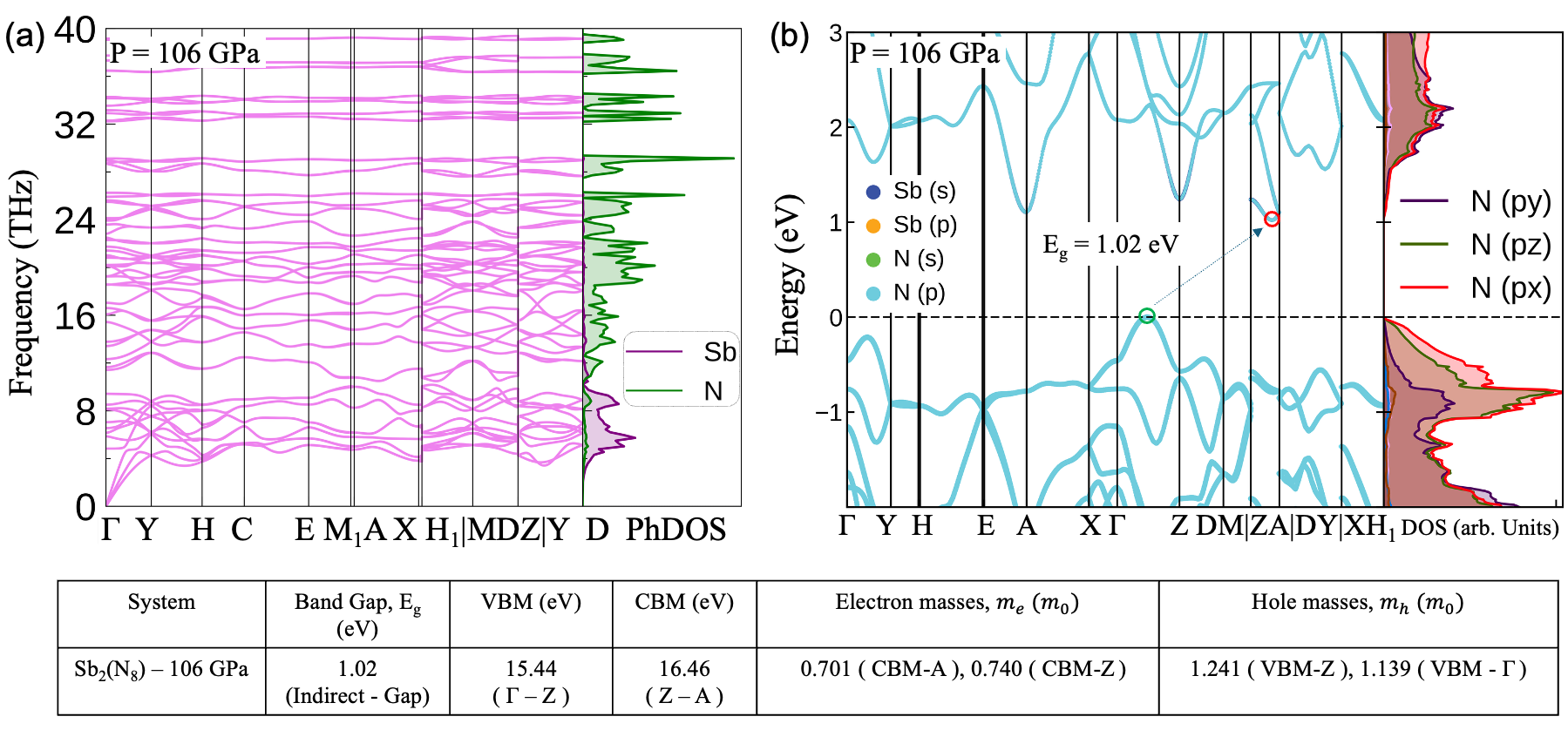


**Figure S5: (a)** Phonon dispersion relations and phonon density of states (PhDOS) for Sb_2_(N_8_) at 106 GPa. **(b)** Electronic band structure and nitrogen *p*-orbital projected electronic density of states (DOS) of Sb_2_(N_8_) at 106 GPa. An indirect semiconducting band gap of 1.02 eV is obtained using the GGA-PBE functional. The table in the bottom panel shows the effective electron/hole masses at the band edges of Sb_2_(N_8_) at 106 GPa.


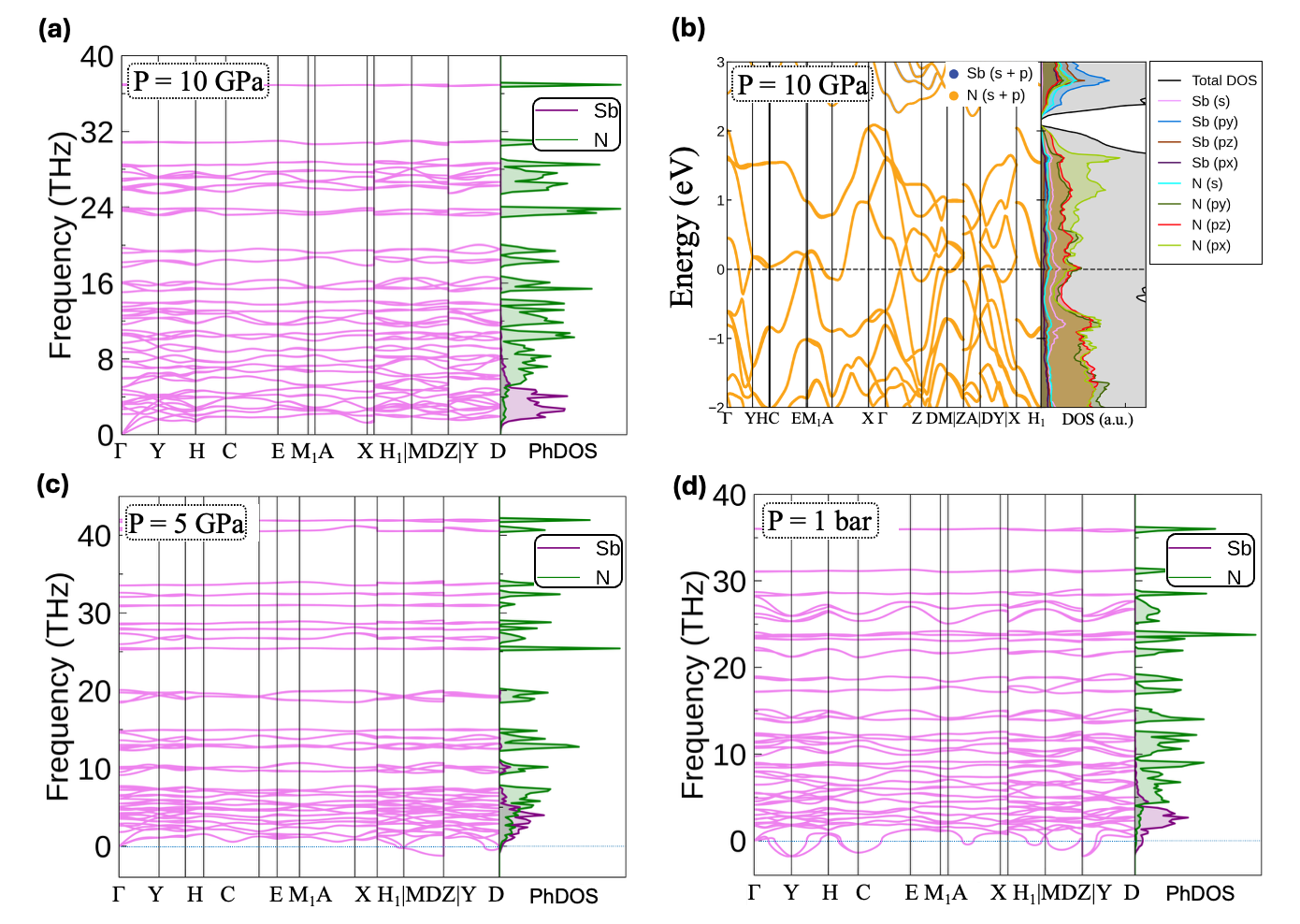


**Figure S6: (a)** Phonon dispersion relations and phonon density of states (PhDOS) for Sb_2_(N_8_) at 10 GPa. The absence of any imaginary phonon branches across the entire Brillouin zone (BZ) indicates the dynamical stability of the compound down to 10 GPa. **(b)** Electronic band structure and atom projected electronic density of states (DOS) of Sb_2_(N_8_) at 10 GPa. **(c)-(d)** Phonon dispersion at calculated pressures of 5 GPa and 1 bar, respectively.


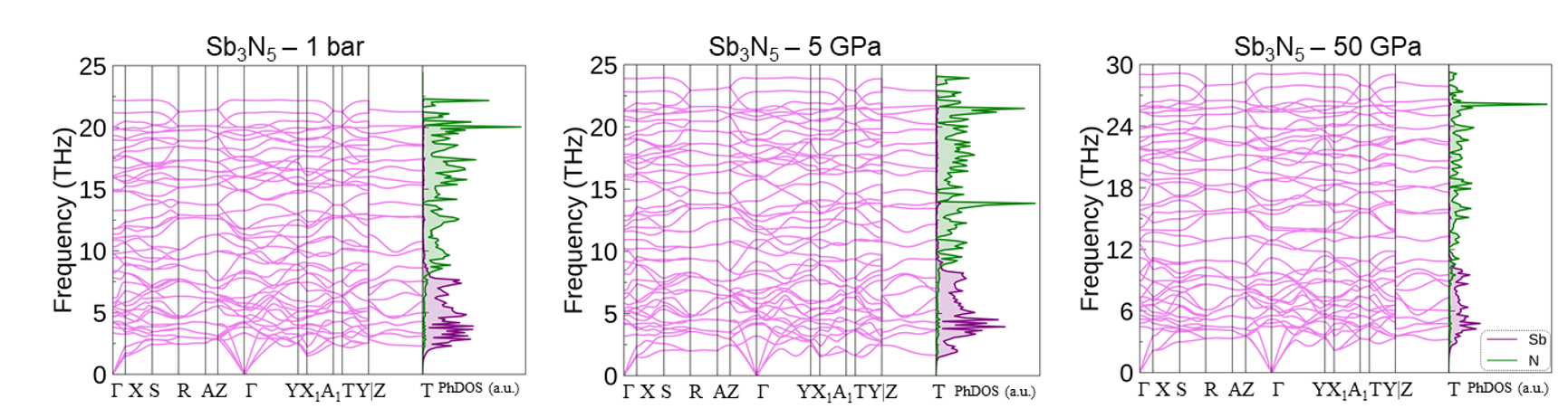


**Figure S7: (a)** Phonon dispersion relations for Sb_3_N_5_ at 1 bar, 5 GPa and 50 GPa, respectively. All calculated vibrational frequencies are real, indicating the dynamic stability of the compound.


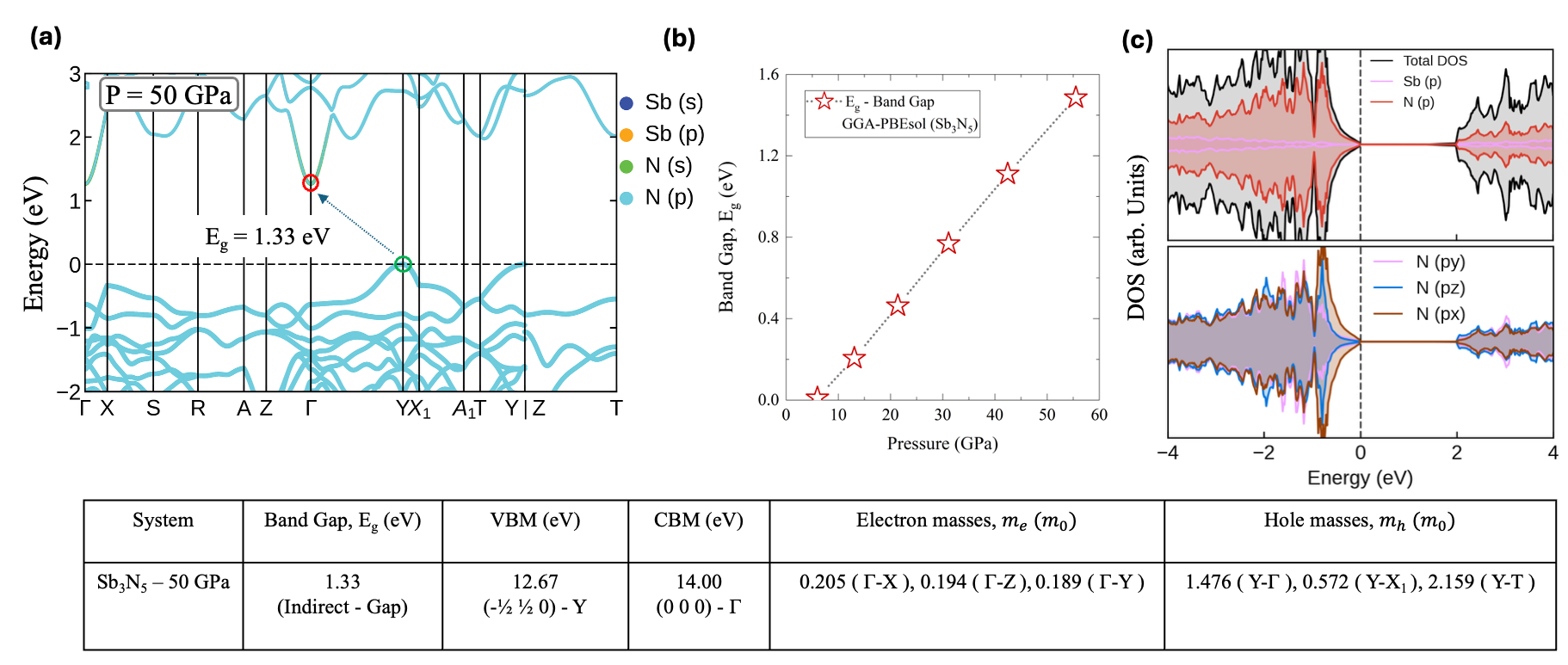


**Figure S8: (a)** Band structure of Sb_3_N_5_ at 50 GPa with an indirect semiconducting band gap of 1.33 eV. **(b)** Variation in fundamental band gap of Sb_3_N_5_ as function of pressure using optimized PBEsol geometries. **(c)** Electronic density of states (DOS) at 50 GPa with element and orbital compositions of DOS. The table in the bottom panel shows the effective electron/hole masses at the band edges of Sb_3_N_5_ at 50 GPa.


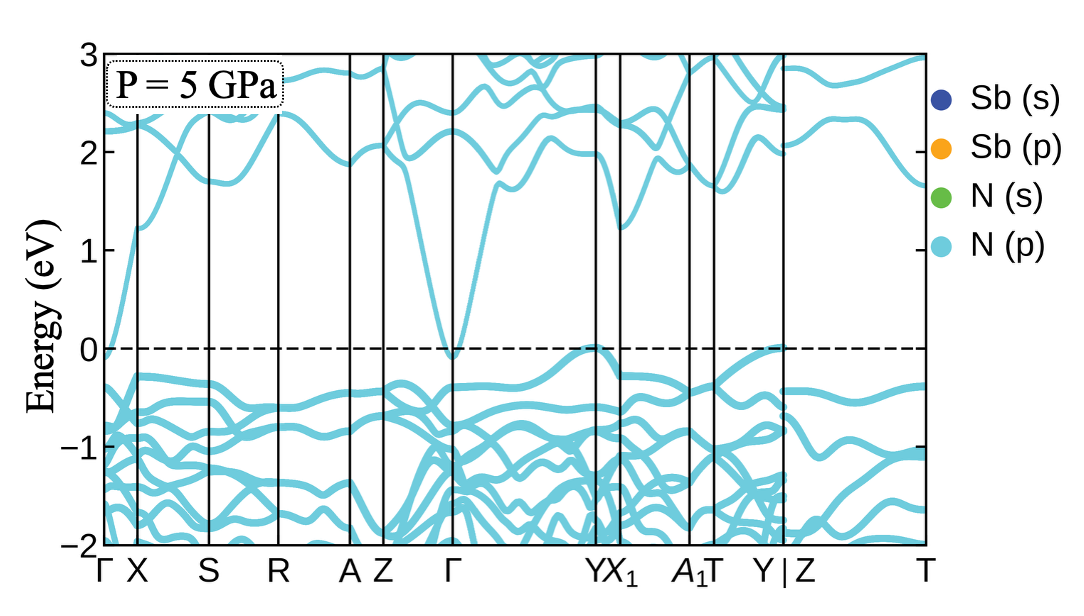


**Figure S9:** Electronic band structure of Sb_3_N_5_ at 5 GPa, obtained using the GGA-PBEsol functional and the corresponding PBEsol relaxed geometry.


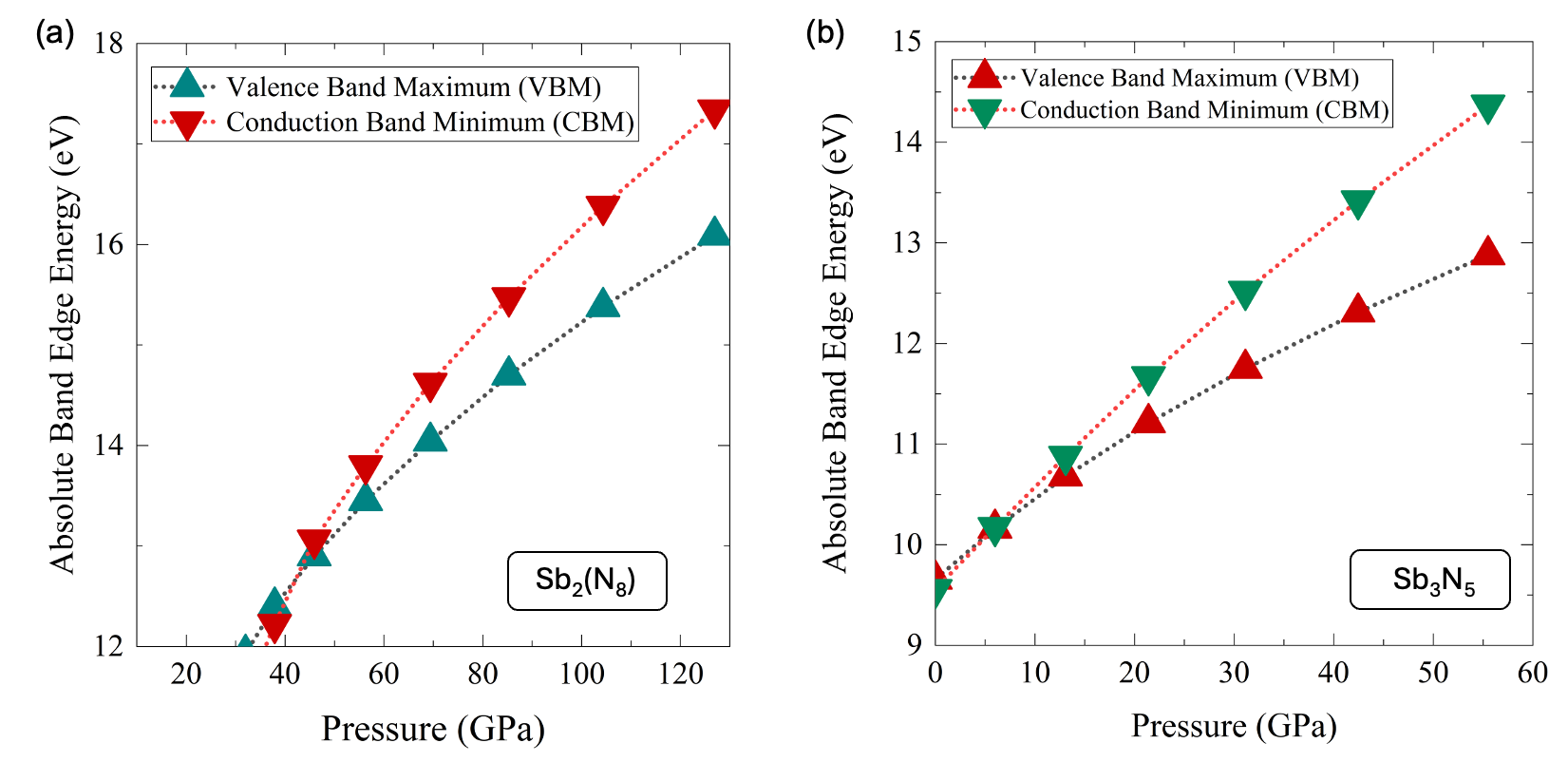


**Figure S10:** Band edge (VBM, CBM) energies as function of pressure showing a semiconductor to metal transition in Sb_2_(N_8_) and Sb_3_N_5_ at different pressure intervals.

# Section F: References

[1] T. Sasaki, K. Shindo, K. Niizeki, “High-pressure phases of group-5B elements: Arsenic and antimony” *Solid State Commun.* **1988**, *67*, 569–572.

[2] H. K. Mao, J. Xu, P. M. Bell, “Calibration of the ruby pressure gauge to 800 kbar under quasi‐hydrostatic conditions” *J. Geophys. Res. Solid Earth* **1986**, *91*, 4673–4676.

[3] A. Dewaele, M. Torrent, P. Loubeyre, M. Mezouar, “Compression curves of transition metals in the Mbar range: Experiments and projector augmented-wave calculations” *Phys. Rev. B* **2008**, *78*, 104102.

[4] C.-S. Zha, W. A. Bassett, S.-H. Shim, “Rhenium, an *in situ* pressure calibrant for internally heated diamond anvil cells” *Rev. Sci. Instrum.* **2004**, *75*, 2409–2418.

[5] G. Shen, V. B. Prakapenka, P. J. Eng, M. L. Rivers, S. R. Sutton, “Facilities for high-pressure research with the diamond anvil cell at GSECARS” *J. Synchrotron Radiat.* **2005**, *12*, 642–649.

[6] H.-P. Liermann, Z. Konôpková, W. Morgenroth, K. Glazyrin, J. Bednarčik, E. E. McBride, S. Petitgirard, J. T. Delitz, M. Wendt, Y. Bican, A. Ehnes, I. Schwark, A. Rothkirch, M. Tischer, J. Heuer, H. Schulte-Schrepping, T. Kracht, H. Franz, “The Extreme Conditions Beamline P02.2 and the Extreme Conditions Science Infrastructure at PETRAIII” *J. Synchrotron Radiat.* **2015**, *22*, 908–924.

[7] E. Bykova, G. Aprilis, M. Bykov, K. Glazyrin, M. Wendt, S. Wenz, H.-P. Liermann, J. T. Roeh, A. Ehnes, N. Dubrovinskaia, L. Dubrovinsky, “Single-crystal diffractometer coupled with double-sided laser heating system at the Extreme Conditions Beamline P02.2 at PETRAIII” *Rev. Sci. Instrum.* **2019**, *90*, 073907.

[8] A. Aslandukov, M. Aslandukov, N. Dubrovinskaia, L. Dubrovinsky, “*Domain Auto Finder (DAFi)* program: the analysis of single-crystal X-ray diffraction data from polycrystalline samples” *J. Appl. Crystallogr.* **2022**, *55*, 1383–1391.

[9] O. V. Dolomanov, L. J. Bourhis, R. J. Gildea, J. A. K. Howard, H. Puschmann, “OLEX2 : a complete structure solution, refinement and analysis program” *J. Appl. Crystallogr.* **2009**, *42*, 339–341.

[10] G. M. Sheldrick, “*SHELXT* – Integrated space-group and crystal-structure determination” *Acta Crystallogr. Sect. Found. Adv.* **2015**, *71*, 3–8.

[11] C. Prescher, V. B. Prakapenka, “*DIOPTAS*: a program for reduction of two-dimensional X-ray diffraction data and data exploration” *High Press. Res.* **2015**, *35*, 223–230.

[12] V. Petříček, M. Dušek, L. Palatinus, “Crystallographic Computing System JANA2006: General features” *Z. Für Krist. - Cryst. Mater.* **2014**, *229*, 345–352.

[13] L. Link, R. Niewa, “Polynator: a tool to identify and quantitatively evaluate polyhedra and other shapes in crystal structures” *J. Appl. Crystallogr.* **2023**, *56*, 1855–1864.

[14] J. Gonzalez-Platas, M. Alvaro, F. Nestola, R. Angel, “EosFit7-GUI : a new graphical user interface for equation of state calculations, analyses and teaching” *J. Appl. Crystallogr.* **2016**, *49*, 1377–1382.

[15] Y. Akahama, H. Kawamura, “Pressure calibration of diamond anvil Raman gauge to 310GPa” *J. Appl. Phys.* **2006**, *100*, 043516.

[16] G. Kresse, J. Furthmüller, “Efficiency of ab-initio total energy calculations for metals and semiconductors using a plane-wave basis set” *Comput. Mater. Sci.* **1996**, *6*, 15–50.

[17] J. P. Perdew, K. Burke, M. Ernzerhof, “Generalized Gradient Approximation Made Simple” *Phys. Rev. Lett.* **1996**, *77*, 3865–3868.

[18] G. Kresse, D. Joubert, “From ultrasoft pseudopotentials to the projector augmented-wave method” *Phys. Rev. B* **1999**, *59*, 1758–1775.

[19] G. Kresse, J. Furthmüller, “Efficient iterative schemes for *ab initio* total-energy calculations using a plane-wave basis set” *Phys. Rev. B* **1996**, *54*, 11169–11186.

[20] J. P. Perdew, A. Ruzsinszky, G. I. Csonka, O. A. Vydrov, G. E. Scuseria, L. A. Constantin, X. Zhou, K. Burke, “Restoring the Density-Gradient Expansion for Exchange in Solids and Surfaces” *Phys. Rev. Lett.* **2008**, *100*, 136406.

[21] H. J. Monkhorst, J. D. Pack, “Special points for Brillouin-zone integrations” *Phys. Rev. B* **1976**, *13*, 5188–5192.

[22] K. Momma, F. Izumi, “*VESTA 3* for three-dimensional visualization of crystal, volumetric and morphology data” *J. Appl. Crystallogr.* **2011**, *44*, 1272–1276.

[23] A. Togo, “First-principles Phonon Calculations with Phonopy and Phono3py” *J. Phys. Soc. Jpn.* **2023**, *92*, 012001.

[24] M. Yu, D. R. Trinkle, “Accurate and efficient algorithm for Bader charge integration” *J. Chem. Phys.* **2011**, *134*, 064111.
